# Aging and Corneal Nerve Health: Mechanisms of Degeneration and Emerging Therapies for the Cornea

**DOI:** 10.3390/cells14211730

**Published:** 2025-11-04

**Authors:** Hanieh Niktinat, Melinda Alviar, Marziyeh Kashani, Hamed Massoumi, Ali R. Djalilian, Elmira Jalilian

**Affiliations:** 1 Department of Ophthalmology and Visual Sciences, Illinois Eye and Ear Infirmary, University of Illinois Chicago, Chicago, IL 60612, USA; niktinat@uic.edu (H.N.); malviar@uic.edu (M.A.); mkasha2@uic.edu (M.K.); hmasso2@uic.edu (H.M.); adjalili@uic.edu (A.R.D.); 2 Richard and Loan Hill Department of Biomedical Engineering, University of Illinois Chicago, Chicago, IL 60607, USA

**Keywords:** aging, corneal nerves, subbasal nerve plexus, ocular surface disease, confocal microscopy, nerve regeneration, extracellular vesicles (EVs), neurodegeneration

## Abstract

Corneal nerves play a crucial role in maintaining ocular surface homeostasis by supporting the functional integrity of corneal epithelial, stromal, and endothelial cells; modulating tear secretion; and facilitating sensory responses essential for overall ocular health. With advancing age, these highly specialized peripheral sensory fibers undergo progressive attrition and morphologic distortion driven by the canonical hallmarks of aging including genomic instability, impaired proteostasis, mitochondrial dysfunction, and chronic low-grade inflammation. The resulting neuro-immune dysregulation reduces trophic support, delays wound healing, and predisposes older adults to dry-eye disease, neurotrophic keratopathy, and postsurgical hypoesthesia. Age-exacerbating cofactors including diabetes, dyslipidemia, neurodegenerative disorders, topical preservatives, chronic contact-lens wear, herpes zoster ophthalmicus, and ocular-surface hypoxia further accelerate sub-basal nerve rarefaction and functional decline. This review provides an overview of age-related physiological alterations in ocular surface nerves, with a particular emphasis on corneal innervation. It also discusses risk factors that speed up these changes. Given the inherently limited regenerative capacity of corneal nerves and their inability to fully restore to baseline conditions following injury or degeneration, it is critical to identify and develop effective strategies aimed at mitigating or delaying physiological nerve degeneration and promoting nerve regeneration. This review also brings up emerging therapeutic strategies, including regenerative medicine, neuroprotective agents, and lifestyle interventions aimed at mitigating age-related corneal nerve degeneration.

## 1. Introduction

In the past five decades, the average lifespan has increased due to high-quality and improved health services [1]. This has led to growing interest in elucidating the mechanisms underlying the aging process. Similar to other biological systems and cellular components, the nervous system and its constituent neurons exhibit structural and functional changes associated with advancing age [2]. Extensive research has been dedicated to understanding age-related alterations in neuronal structures. Notably, the cornea possesses the highest density of nerves among human tissues, exhibiting a nerve density approximately 300–600 times greater than that of the skin [3].

**Figure 1:**
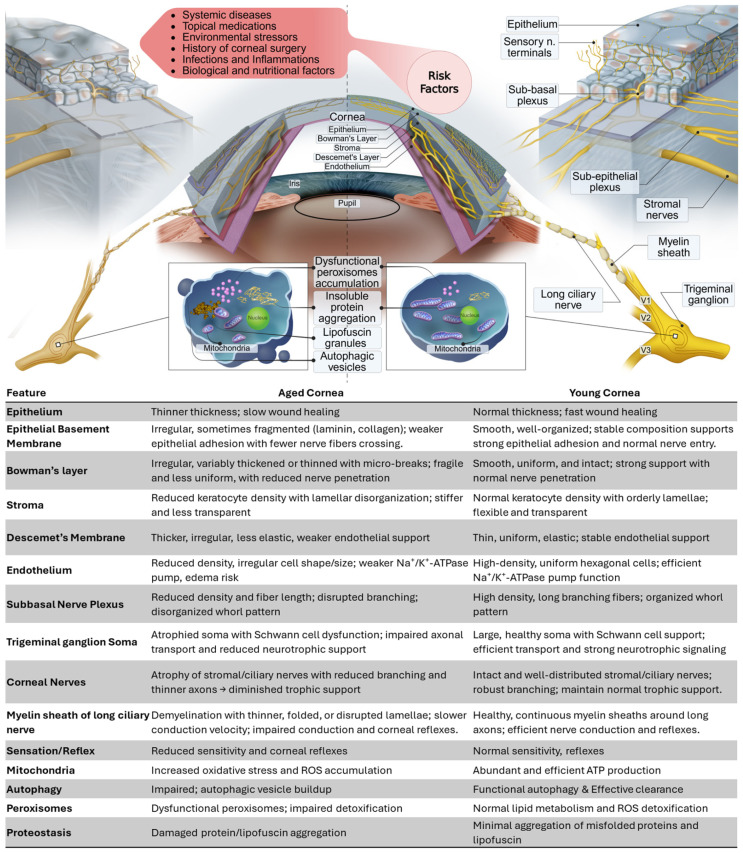
Structural and functional differences between aged and young cornea. Illustration and summary table depicting age-related alterations in corneal layers, cellular compartments, and neuronal structures. Aged corneas exhibit epithelial thinning, irregular basement membrane, reduced nerve density, mitochondrial and peroxisomal dysfunction, impaired proteostasis, and delayed wound healing, in contrast to the intact architecture and efficient repair capacity of young corneas.

Corneal innervation is derived from the ophthalmic branch of the trigeminal nerve, where corneal nerve fibers enter the cornea through the limbal corneoscleral junction and arborize extensively within the anterior stroma. These stromal nerve branches give rise to the subepithelial nerve plexus, a dense network located just beneath Bowman’s layer. Importantly, nerve fibers do not extend into the deeper stromal layers adjacent to Descemet’s membrane or the corneal endothelium. The subepithelial plexus exhibits a higher nerve density in the peripheral cornea compared to the central region [4]. From the subepithelial plexus, finer nerve fibers penetrate Bowman’s layer and enter the basal epithelial layer, where they form the subbasal nerve plexus. In contrast to the subepithelial plexus, the subbasal nerve plexus along with intraepithelial branches and superficial nerve terminals shows significantly greater density in the central cornea. This region-specific organization of corneal innervation is critical for maintaining epithelial homeostasis and optimizing sensory function within the visual axis [5,6]. However, no significant differences in corneal nerve density or nerve fiber counts have been observed among the four corneal quadrants, suggesting a relatively uniform circumferential distribution (Figure 1) [6].

The normal function and structural integrity of corneal nerves are essential for maintaining the health and regenerative capacity of corneal epithelial and endothelial cells, supporting basal tear secretion, and safeguarding the ocular surface against chemical, thermal, and nociceptive insults. Disruption or dysfunction of corneal innervation can impair neurotrophic support and compromise ocular surface homeostasis, contributing to the development of conditions such as neurotrophic keratitis [7,8]. The incidence of neurotrophic keratitis is higher in individuals over the age of 60, largely due to the increased prevalence of risk factors that contribute to corneal nerve damage [8]. These include diabetes mellitus, herpetic infections, glaucoma, a history of ocular surgery, chronic use of topical medications, dry eye disease, and long-term contact lens wear. Collectively, these factors can accelerate age-related degeneration of corneal nerves, impairing neurotrophic support and increasing susceptibility to ocular surface disease [9,10,11,12,13,14,15,16]. In elderly individuals, corneal nerve impairment contributes to the pathogenesis of dry eye disease by reducing basal tear secretion and disrupting reflexive blinking. This neurogenic dysfunction exacerbates ocular surface desiccation and inflammation, leading to symptoms such as ocular pain, foreign body sensation, photophobia, excessive tearing, and a persistent burning sensation.

Multiple studies have consistently demonstrated that corneal nerve parameters, such as nerve density and nerve fiber length, as well as corneal sensitivity, progressively decline with advancing age. Given the inherently limited regenerative capacity of corneal nerves and their inability to fully restore structural and functional properties following degeneration or injury, there is a pressing need to elucidate the underlying molecular and cellular mechanisms responsible for age-related corneal nerve alterations. Such understanding is crucial to developing targeted strategies aimed at delaying or mitigating these degenerative processes or enhancing nerve regeneration therapeutically [17]. In this review, we analyze the physiological changes occurring in corneal nerves with aging, summarizing and interpreting the findings from key studies addressing these age-associated alterations. Additionally, we discuss the current understanding of mechanisms underlying corneal nerve degeneration and highlight emerging therapeutic interventions intended to delay nerve aging or stimulate effective regeneration. All chemical compounds discussed in this study are listed in Supplementary Table S1 along with their corresponding chemical structures, mechanisms, and relevant details.

## 2. Pathophysiological Changes Associated with Aging

### 2.1. Physiology of Aging and Cellular Changes

Aging is characterized by a progressive loss of physiological integrity, leading to functional decline and increased vulnerability to disease [18]. This decline is driven by multiple interconnected cellular and molecular mechanisms collectively termed the “hallmarks of aging.” As outlined by López-Otín et al., these include: genomic instability, telomere attrition, epigenetic alterations, loss of proteostasis, deregulated nutrient sensing, mitochondrial dysfunction, cellular senescence, stem cell exhaustion, altered intercellular communication, and more recently recognized features such as impaired macroautophagy, chronic inflammation, and gut microbiome dysbiosis [19]. Initiating events such as DNA damage, epigenetic drift, and proteostasis collapse impair tissue homeostasis. Hyperactive nutrient-sensing pathways (e.g., IGF-1/mTOR) further accelerate aging, while mitochondrial dysfunction contributes to oxidative stress and energy deficits. Impaired autophagy leads to the accumulation of damaged organelles, and chronic sterile inflammation (“inflamm-aging”) disrupts tissue function systemically. Dysbiosis of the gut microbiome exacerbates these effects by promoting inflammation and metabolic imbalance [20].

Among these hallmarks, cellular senescence is particularly notable. Triggered by DNA damage, telomere shortening, oncogene activation, or mitochondrial stress, senescence entails permanent cell-cycle arrest. While transient senescence plays protective roles in tissue repair and tumor suppression, chronic senescent cell accumulation driven by declining immune clearance contributes to tissue dysfunction. These cells secrete a pro-inflammatory mix of cytokines, proteases, and growth factors known as the senescence-associated secretory phenotype (SASP), which perpetuates extracellular matrix remodeling and low-grade inflammation [21,22]. Chronic senescence burden contributes to neurodegeneration, cardiovascular pathology, cancer, and vision-threatening eye disease [23]. Therapeutic strategies aimed at modulating this balance using senolytics, SASP inhibitors, or autophagy enhancers are emerging as promising approaches to mitigate age-related pathology both systemically and within ocular tissues. Collectively, these hallmarks highlight the multifactorial and interconnected nature of aging and provide a framework for developing targeted anti-aging interventions.

In ocular tissues, cumulative oxidative and environmental stress induces cellular senescence in the corneal epithelium/endothelium, retinal pigment epithelium, and trabecular meshwork; the resulting SASP-driven inflammation and matrix remodelling promote drusen formation in age-related macular degeneration [24], elevate intraocular pressure in glaucoma [25], and accelerate endothelial cell loss in Fuchs dystrophy, transforming a transiently protective response into a driver of disease progression [26,27]. Understanding the dual role of senescence, protective in youth but pathological with age, offers new therapeutic opportunities, including senolytics and SASP modulators, to preserve ocular health and mitigate age-related vision loss [26].

### 2.2. Aging in Peripheral and Central Nervous Systems

In both the peripheral and central nervous systems, aging induces structural, functional, and cellular changes that compromise nerve integrity and functionality. In the peripheral nervous system (PNS), common age-related alterations include reductions in nerve fiber density, axonal atrophy, and demyelination, which contribute to impaired nerve regeneration and slower conduction velocity [2,28,29,30]. Importantly, unmyelinated axons which are present abundantly in corneal nerve endings are more vulnerable to aging, exhibiting greater losses in number and density compared to myelinated axons [28]. Myelinated fibers also undergo significant morphological changes, such as lamellar disruptions, myelin folds, and the formation of notches that can lead to axonal atrophy [29]. In aging individuals, the accumulation of intracellular waste products such as lipofuscin granules and dysfunctional mitochondria can further compromise nerve function [31].

Age-related alterations also extend to neuronal dendrites, characterized by reductions in dendritic length, branching complexity, and dendritic spine density, collectively impairing synaptic connectivity and neuronal function [32]. While some neuronal populations retain their synaptic structural integrity during aging, CNS-resident glia—particularly microglia (CNS immune cells) and astrocytes (CNS support cells)—progressively adopt a pro-inflammatory phenotype. This shift contributes to chronic, low-grade neuroinflammation, thereby impeding neuronal repair and regeneration [33]. Additionally, mitochondrial dysfunction and increased oxidative stress, hallmarks of neuronal aging, exacerbate disruptions in axonal transport, compromise energy metabolism, and reduce neuronal viability in both the CNS and PNS [31,34].

In both sensory and autonomic ganglia, age-associated changes are not limited to neurons but also affect supportive glial populations. Schwann cells (myelinating glia of the PNS), which play a central role in axonal maintenance and regeneration in the peripheral nervous system (PNS), exhibit diminished responsiveness with age, including reduced production of neurotrophic factors, impaired phagocytic activity, and decreased capacity to support axonal repair. These deficits contribute to delayed nerve regeneration, often resulting in smaller-caliber axons and thinner myelin sheaths [2,35,36]. Additionally, aging Schwann cells may enter a senescent state, characterized by a pro-inflammatory secretory profile that exacerbates local inflammation and further impairs remyelination processes [37]. Similarly, satellite glial cells (SGCs; non-myelinating glia of PNS sensory ganglia) undergo age-related functional alterations, including disrupted calcium signaling and up-regulated inflammatory mediators, thereby contributing to the decline in peripheral-nerve homeostasis [38].

The autonomic nervous system (part of the PNS) also experiences age-related functional decline, manifesting as reduced heart rate variability, impaired thermoregulatory control, and diminished sympathetic nerve regenerative capacity all of which progressively worsen with advancing age [39]. In parallel, the age-associated decline in the expression of critical neurotrophic factors, such as brain-derived neurotrophic factor (BDNF), further compromises the structural integrity and function of peripheral nerves [40]. Collectively, these multifaceted alterations highlight the systemic nature of neural aging and provide a mechanistic framework for understanding analogous degenerative processes in ocular nerves including cornea, retinal and optic nerves, which experience significant age-related structural and functional changes.

Although retinal and optic nerve aging is not the primary focus of this review, a brief overview is included to contextualize corneal nerve degeneration within the broader framework of ocular and neural aging. Retinal ganglion cells (RGCs) and their axons, which form the optic nerve, undergo progressive age-related alterations including axonal loss, mitochondrial dysfunction, and increased oxidative stress [41,42,43]. These changes predominantly affect small-caliber fibers, which are more metabolically vulnerable [43]. Optical coherence tomography (OCT) studies have consistently reported age-related thinning of the retinal nerve fiber layer (RNFL), often exceeding the rate of RGC loss, likely due to contributions from non-neuronal components [44,45,46,47,48,49,50]. Aging also impairs oligodendrocyte function and myelin maintenance in the post-laminar optic nerve, leading to reduced conduction speed and contributing to axonal degeneration [51]. Furthermore, aging activates glial cells—including astrocytes, microglia, and Müller cells—toward a pro-inflammatory state, increasing cytokine and reactive oxygen species (ROS) production and promoting RGC apoptosis [52].

Age-related neural changes may contribute to postoperative pain following vitreoretinal surgery [53,54]. While variability in measurement techniques and individual differences in axonal diameter may account for discrepancies in the literature regarding the extent of optic nerve aging [55,56], the overall pattern supports a decline in structural and functional integrity. These mechanisms, oxidative stress, neuroinflammation, glial reactivity, and axonal loss, mirror those observed in the aging cornea. Thus, insights from posterior ocular nerve aging support the development of shared therapeutic strategies. The remainder of this review will focus specifically on age-related changes in corneal nerves and emerging interventions to preserve their structure and function.

## 3. Impact of Aging on Corneal Nerves

### 3.1. Structural and Functional Changes in Corneal Nerves with Age

Corneal nerve development begins at birth, with a progressive increase in the number and density of subbasal and epithelial nerves as the corneal surface expands. This postnatal maturation supports corneal homeostasis, sensory function, and epithelial integrity [57]. However, aging induces structural and functional changes in corneal nerves, including reductions in subbasal nerve density, superficial nerve terminal length, and vertical connecting nerves, which compromise corneal sensitivity and epithelial maintenance [58,59]. While most studies report a progressive decline in corneal nerve parameters with age, variations exist due to differences in study design and methodologies [5,6,57,58,59,60,61,62,63,64,65,66,67]. The mean corneal subbasal nerve density (SND), measured via in vivo confocal microscopy (IVCM), is approximately 19 mm/mm^2^ in healthy individuals and declines annually by 0.25% to 0.30% [61]. Taurone et al. found that thin nerve fibers are preferentially lost, while thicker fibers are relatively preserved, leading to a progressive decline in corneal sensitivity, particularly after age 70. This reduction begins in the peripheral cornea and progresses centrally, highlighting regional vulnerability [60]. In contrast, Erie et al. conducted a study examining the effect of age on the corneal subbasal nerve plexus. They assessed 65 healthy subjects aged 15–79 and measured metrics such as subbasal nerve density, number, and orientation. Their findings showed no significant correlation between age and subbasal nerve density or orientation [68]. Furthermore, confounding factors such as systemic diseases such as type 2 diabetes can further exacerbate age-related nerve changes [69]. Taken together, these findings emphasize the complex interplay between intrinsic (chronological aging) and extrinsic (environmental and health-related) influences on corneal nerve morphology. Additionally, they underscore the potential of large-scale imaging to clarify the timeline of corneal nerve degeneration, support earlier detection of nerve pathologies, and guide targeted therapeutic strategies in aging populations.

Conflicting evidence exists regarding age-related changes in corneal nerve tortuosity. While Roszkowska et al. observed age-dependent declines in most corneal nerve parameters, with the exception of nerve beading density (NBD) [62], other studies reported that tortuosity remains unchanged with advancing age [67,70]. In contrast, Tavakoli et al. described an increase in nerve tortuosity associated with aging [71]. Additionally, aging has been shown to decrease inferior corneal whorl nerve length (IWL) at a rate of 0.2088 mm/mm^2^ per year, a phenomenon implicated in the decline of corneal sensitivity [63]. Conversely, Chin et al. reported that nerve fiber area, width, and branch density remain stable after the age of 65 [64] and other investigations found no significant age-related changes in corneal nerve branching or splitting patterns [67,71].

Despite preserved axonal thickness, functional decline in corneal nerves with aging impairs corneal sensitivity and epithelial maintenance. These functional deficits may result from age-associated reductions in the expression of mRNAs encoding proteins crucial for axonal growth, thereby disrupting nerve regeneration and homeostasis [59]. Corneal nerve loss also influences the health and function of both endothelial and epithelial cells; however, the sequence of these changes—whether nerve alterations precede epithelial changes or vice versa—remains to be elucidated. Nonetheless, it is well established that decreased nerve density significantly compromises the integrity of the corneal surface, contributing notably to the pathogenesis of neurotrophic keratitis [60].

Age-related corneal sensitivity loss progresses from the peripheral toward the central cornea, a pattern likely attributable to nerve degeneration, diminished trophic support, and impaired regenerative capabilities [72]. As corneal sensitivity decreases, the responsiveness of cold-sensitive receptors essential modulators of tear production and blinking reflexes also diminishes. This reduction further exacerbates dry eye disease and elevates the risk of ocular surface damage [73]. Interestingly, despite the decline in corneal nerve density and sensitivity, neuronal–epithelial interactions appear to increase with age, a phenomenon possibly associated with autophagosome accumulation at nerve–epithelial fusion sites. Although this enhanced interaction may serve a compensatory role in supporting epithelial maintenance following nerve loss, the precise contribution of this mechanism within the aging cornea remains an active area of investigation [74].

In contrast to corneal nerves, the dendritic cells within the cornea, the key mediators of immune surveillance, do not exhibit significant age-related alterations, suggesting that corneal nerve degeneration proceeds independently of immune alterations [58,75]. Aging notably influences the spatial arrangement of inferocentral subbasal nerves, transitioning from a symmetrical, clockwise orientation in young individuals to a diminished and less organized pattern in older adults. Although systemic conditions such as type 2 diabetes mellitus (T2DM) and Parkinson’s disease (PD) further modify corneal nerve architecture, the nerve whorl patterns in these diseases closely resemble those observed in age-matched healthy individuals. This finding underscores aging itself as the predominant factor driving corneal nerve architectural changes, rather than disease-specific mechanisms [76].

Studies assessing age-related changes in corneal nerves report varying findings. While some studies indicate stability in corneal nerve number and density with age, others specifically report increased nerve tortuosity, a separate morphological characteristic describing nerve curvature and complexity [77,78]. Discrepancies across studies on age-related changes in corneal nerves often stem from differences in age-range selection and methodological variations. Specifically, research focused exclusively on participants younger than approximately 60 years typically reports minimal to no significant changes in nerve number, density, arrangement, or beading. In contrast, comprehensive studies including subjects beyond the sixth decade of life (≥60 years), and especially those aged ≥65 years, consistently show a significant age-related reduction in corneal nerve fiber length and density [64]. These findings underscore the importance of considering participant age and standardized methodologies when interpreting data on age-related corneal nerve alterations [68,79]. Despite relatively stable nerve density observed in some aging studies, corneal sensitivity frequently declines with advancing age. This paradox may arise from damage to the small, free nerve endings within the corneal epithelium, structures typically undetectable by standard in vivo confocal microscopy (IVCM). Moreover, subbasal nerve bundles encompass multiple axons, allowing functional deficits at the individual axonal level such as reduced axonal conduction or impaired neuronal signaling to diminish sensory responsiveness without producing apparent structural abnormalities. Additional age-related changes at nuclear and synaptic levels within the corneal nerves may further compromise sensory function. Together, these findings highlight the critical need for integrating both manual and automated analytical techniques to enhance the sensitivity and accuracy of age-related corneal nerve assessments [68].

### 3.2. Methodological Considerations in Corneal Nerve Aging Studies

Variability in reported outcomes of corneal nerve aging studies arises from numerous methodological factors, including differences in the selected age ranges of study populations, specific corneal layers evaluated (e.g., subbasal, subepithelial, or terminal epithelial nerves), and distinct nerve parameters quantified (total nerve length, density, or number). Additionally, the choice of imaging modality significantly influences findings; slit-scanning and tandem-scanning confocal microscopy methods frequently fail to detect subtle age-related nerve changes, whereas fluorescence-based and laser-scanning confocal microscopy techniques demonstrate clear reductions in nerve density with advancing age [80,81]. Further methodological variability is introduced by differences in study protocols. These include the field-of-view dimensions, selected anatomical regions for imaging, the number of images acquired per participant, examiner expertise levels, and the randomization strategies used. Given that in vivo confocal microscopy (IVCM) captures only approximately 0.15% of the total corneal surface area [63], these protocol differences can substantially impact outcomes. Moreover, analytical techniques whether manual or automated [61] and variations in immunostaining methodologies, including choice of neuronal markers (such as β3-tubulin, Substance P (SP), or Calcitonin Gene-Related Peptide (CGRP)), further contribute to inconsistent results across studies. Therefore, standardized imaging and analytical protocols are essential to enhance reproducibility and comparability among studies examining age-related changes in corneal nerves.

## 4. Risk Factors That Accelerate Age-Related Corneal Nerve Dysfunction

Under baseline physiologic conditions, age-related loss of the corneal sub-basal nerve plexus is relatively modest; however, diverse extrinsic and intrinsic stressors can accelerate this process, yielding structural deficits and markedly diminished corneal sensitivity that far exceed those attributable to chronological ageing alone. Some of these factors include surgical interventions, systemic diseases, chronic inflammation, medication usage, and environmental stressors. Each of these elements uniquely accelerates or exacerbates the intrinsic age-related deterioration of corneal nerve structure and function.

### 4.1. Refractive Surgery Differentially Affects Corneal Nerves and Ageing Slows Their Recovery

Refractive surgeries cause significant corneal nerve damage, with each procedure showing distinct patterns of nerve loss and regeneration. Photorefractive keratectomy (PRK), which removes the epithelium and anterior stroma, transects subbasal nerves while sparing deeper stromal nerves. Subbasal nerve density significantly declines after surgery but recovers to near preoperative levels by 2 years, remaining stable through 5 years. In laser in situ keratomileusis (LASIK), creation of a corneal flap severs a larger fraction of corneal nerves, resulting in slower and more incomplete reinnervation—subbasal nerve fiber density remains ~30–50% below baseline at 1 year and only approaches pre-surgery levels by about 5 years [82]. Even a decade after LASIK, patients show persistently reduced main nerve fiber densities and branching compared to unoperated eyes (indicating incomplete structural recovery), although nerve fiber length and orientation (tortuosity) may normalize with time [83]. Small-incision lenticule extraction (SMILE), which avoids a large flap, tends to spare more nerves and thus exhibits intermediate outcomes: early postoperative studies demonstrate higher residual subbasal nerve density, more intact long nerve fibers, and better corneal sensation in SMILE eyes versus LASIK eyes 6 months post-surgery [84]. Long-term, SMILE also shows superior nerve regeneration compared to LASIK—at ~4–5 years post-op, SMILE-treated corneas have significantly greater subbasal nerve fiber length, density, and branching than LASIK-treated corneas, though notably neither procedure restores innervation to normal age-matched levels by this time [85]. Advancing age is associated with a pronounced delay in corneal nerve recovery after refractive surgery. Older patients have lower baseline corneal nerve density and exhibit slower nerve regrowth and sensory return postoperatively. For example, in a large cohort undergoing SMILE, young adults (around 20–30 years) fully regained pre-surgery central nerve fiber length by 1 year, whereas middle-aged patients (≈40–50 years) had significantly incomplete nerve regeneration at 1 year (with subbasal nerve fiber length, density, and branch density remaining well below preoperative values) [70]. This age-related impairment in nerve regeneration likely extends to LASIK and PRK as well, contributing to more persistent corneal hypoesthesia and dry eye symptoms in older individuals. In summary, refractive surgery induces lasting alterations in corneal innervation characterized by reduced subbasal nerve fiber density and altered morphology. PRK generally permits faster nerve reinnervation than LASIK, and SMILE causes less initial denervation than LASIK, yet all three surgeries can produce prolonged nerve fiber loss and delayed functional recovery of the cornea’s innervation, especially in older corneas.

### 4.2. Systemic Metabolic Disorders Accelerate the Age-Related Loss of Corneal Nerves

Systemic diseases, especially diabetes mellitus and related metabolic disorders, significantly compound age-related corneal nerve dysfunction. In vivo confocal microscopy (IVCM) consistently shows that diabetes mellitus reduces corneal nerve–fiber length and density by 30–60%, increases tortuosity, and predicts the onset and progression of diabetic peripheral neuropathy; tight glycaemic control or bariatric surgery can partially reverse these changes [86,87]. Beyond hyperglycaemia, metabolic syndrome and isolated dyslipidaemia independently aggravate small-fiber damage: individuals with hyper-triglyceridaemia or metabolic syndrome exhibit significantly greater corneal nerve loss and higher neuropathy risk than metabolically healthy peers [88]. Recent data further indicate that obesity exerts an additive adverse effect on corneal nerves in diabetic patients, even after adjusting for HbA1c and blood pressure [89]. These findings implicate systemic inflammation, oxidative stress, and microvascular dysfunction as convergent mechanisms linking metabolic derangement to accelerated corneal neurodegeneration with advancing age.

### 4.3. Corneal Infections and Chronic Inflammatory Disorders Accelerate Age-Related Corneal Neurodegeneration

Corneal infections and chronic inflammatory conditions, particularly herpes zoster ophthalmicus (HZO), represent critical risk factors for accelerated nerve damage [90]. In vivo confocal microscopy (IVCM) studies of herpes zoster ophthalmicus (HZO) demonstrate significant and often bilateral reductions in sub-basal corneal nerve density, which can persist for months to years after the acute eruption. While age-related differences have not been consistently quantified, older patients may experience slower or incomplete nerve regeneration. Varicella-zoster-virus latency in trigeminal ganglia triggers recurrent axonal injury and a pro-neurotoxic cytokine milieu, thereby compounding the intrinsic vulnerability of ageing corneal nerves [91]. Similar patterns of small-fibre loss are reported in herpes simplex keratitis, where repeated epithelial/stromal inflammation leads to microneuroma formation and chronic ocular pain [92]. Systemic autoimmune diseases add a second hit: patients with Sjögren’s syndrome show a stepwise decline in nerve-fibre length and density beginning in the sixth decade, correlating with tear-film hyperosmolarity and the severity of ocular surface damage [93]. Rheumatoid arthritis and systemic lupus erythematosus display comparable corneal nerve rarefaction, attributable to circulating auto-antibodies, complement activation, and persistent ocular-surface inflammation [94,95]. Collectively, these infectious and autoimmune insults impose chronic neuro-inflammatory stress that synergises with ageing to hasten corneal sensory loss, underscoring the need for early antiviral therapy and aggressive immunomodulation to preserve corneal innervation in older adults.

### 4.4. Medication-Associated Neurotoxicity Compounds Age-Related Corneal Nerve Loss

In vivo–confocal-microscopy (IVCM) studies show that long-term instillation of BAK-preserved antiglaucoma drops (e.g., timolol, latanoprost) reduces corneal nerve-fiber density by 25–50%, increases tortuosity, and elevates dendritic-cell infiltration changes largely absent with preservative-free formulations or after medication withdrawal [96,97]. Systemic neurotoxic chemotherapeutics add a second hit: patients receiving oxaliplatin or paclitaxel exhibit a 30–40% decline in corneal nerve-fiber length within three to six treatment cycles, and greater cumulative doses predict more severe nerve loss and corneal hypoesthesia [98,99]. Emerging evidence also implicates chronic opioid, gabapentinoid, and antidepressant use in small-fiber degeneration detectable by IVCM, mirroring the drug-induced peripheral neuropathies seen systemically [100]. Collectively, these findings highlight topical preservatives and systemic neuroactive agents as modifiable accelerants of corneal neurodegeneration in older adults, underscoring the importance of preservative-free ocular therapy and vigilant monitoring of high-risk systemic regimens.

### 4.5. Corneal Nerve Loss as a Biomarker in Age-Related Neurodegenerative Diseases

In vivo confocal microscopy (IVCM) studies in humans (with supporting animal model evidence) consistently demonstrate that major age-related neurodegenerative diseases—including Parkinson’s disease (PD), Alzheimer’s disease (AD) and other dementias, and multiple sclerosis (MS)—are accompanied by significant loss of corneal subbasal nerve fibers. For example, patients with PD and MS both exhibit marked reductions in corneal nerve fiber density (with loss of nerve branches and fiber length) on IVCM, and this corneal neurodegeneration correlates with greater clinical severity and disease progression [101]. Similarly, individuals with AD and other forms of dementia show significantly diminished corneal nerve densities, even at early stages such as mild cognitive impairment [102]. Collectively, these findings indicate that corneal nerve degeneration mirrors the widespread small-fiber neuropathy of central neurodegenerative disorders, underscoring the potential of corneal nerves as accessible biomarkers of neurological decline with aging. Notably, corneal nerve loss has also been observed in emerging conditions like amyotrophic lateral sclerosis (ALS), and ongoing research is exploring similar corneal changes in Huntington’s disease, highlighting the broad translational utility of IVCM in neurodegeneration research [103].

### 4.6. Biological, Reproductive, and Nutritional Factors Modulate Age-Related Corneal Neurodegeneration

Multiparity and the hormonal milieu of pregnancy trigger systemic surges in neurotrophins; serum NGF rises several-fold across gestation and are associated with transient improvements in corneal sensitivity, suggesting a trophic reserve that may help sustain axonal integrity later in life [104,105]. Conversely, micronutrient deficits accelerate nerve loss. Clinical and experimental studies show that vitamin-B12 depletion produces small-fiber neuropathy detectable in the corneal sub-basal plexus, whereas oral or topical mecobalamin restores nerve length and corneal sensitivity within weeks [106,107,108]. Vitamin-D receptor knockout or dietary deficiency similarly reduces corneal nerve density and delays epithelial healing; these effects are partially reversed by active-vitamin-D supplementation [109]. Long-chain omega-3 polyunsaturated fatty acids, especially DHA, enhance axon regeneration after surgery and in diabetic models, and randomized trials report that systemic or topical n-3 therapy augments corneal nerve fiber density within three months [110]. Together, these observations highlight reproductive endocrinology and targeted micronutrient support as modifiable levers for preserving corneal innervation in the aging eye.

### 4.7. Ocular Surface Hypoxia and Environmental Stressors Exacerbate Age-Related Corneal Neurodegeneration

Experimental hypoxia models show that diminished oxygen tension, whether from age-related reductions in corneal perfusion or long-term low-Dk contact-lens wear, induces subbasal nerve beading, truncation, and loss of length on in vivo confocal microscopy (IVCM) [111,112]. Meibomian-gland dysfunction (MGD) further accelerates nerve attrition: patients with evaporative dry eye secondary to MGD display a 25–40% drop in nerve-fiber density and branch density, proportional to gland atrophy and ocular-surface inflammation [113,114]. Exogenous insults add an additional burden: murine and human studies of radiation keratopathy document months-long, >50% nerve loss accompanied by dendritic-cell infiltration, while brief UV-C exposure can obliterate the entire subbasal plexus before slow, incomplete recovery [115,116,117]. These data underscore how hypoxia, inflammatory evaporative stress, ionizing radiation, and ultraviolet light synergistically erode corneal innervation across the lifespan, highlighting the need for oxygen-permeable optics, aggressive MGD therapy, and photoprotection to preserve sensory function in the aging eye.

Collectively, these diverse influences intricately interact with the intrinsic aging processes of corneal nerves, emphasizing the necessity of targeted therapeutic strategies designed to mitigate nerve loss, preserve corneal sensitivity, and maintain ocular surface integrity in older adults.

## 5. Molecular Mechanisms of Corneal Nerve Aging

Aging involves complex molecular and cellular alterations that collectively undermine corneal nerve integrity. Multiple interrelated mechanisms drive corneal nerve degeneration, notably oxidative stress, metabolic disturbances, inflammation, mitochondrial dysfunction, and impaired neurotrophic signaling. These changes culminate in DNA damage, genomic instability, decreased regenerative capacity, and heightened neuronal loss, ultimately manifesting as corneal nerve thinning, reduced nerve density, and functional deterioration [34]. This section outlines critical molecular pathways central to corneal nerve aging, emphasizing oxidative stress, disrupted energy metabolism, and immune-mediated inflammation.

### 5.1. Free Radical Oxidative Stress and ROS Accumulation

Aging is characterized by a progressive increase in reactive oxygen species (ROS) and lipid peroxidation, leading to cumulative oxidative damage in nucleic acids, proteins, and lipids [118]. ROS-induced mitochondrial dysfunction plays a key role in corneal nerve aging, disrupting axonal metabolism and increasing susceptibility to neurodegeneration [34,118]. Aging corneal nerves exhibit diminished antioxidant defenses, exacerbating oxidative stress and accelerating nerve deterioration [119].

#### 5.1.1. NGF/TrkA Signaling Impairment

Nerve Growth Factor (NGF) regulates neurite outgrowth, branching, and sprouting, playing a key role in corneal nerve regeneration. [120] Age-related reductions in NGF expression and decreased activation of its high-affinity receptor, tropomyosin-related kinase A (TrkA), impair nerve repair processes. Elevated ROS levels further disrupt NGF signaling by reducing proNGF and TrkA availability [64,121]. Given the critical role of NGF-TrkA interactions in nerve regeneration and morphological complexity, reduced NGF-TrkA signaling contributes significantly to shorter and less dense nerve fibers in aging corneas [64].

#### 5.1.2. Substance P and Inflammatory Regulation

Substance P (SP) is a neuropeptide crucial for maintaining corneal nerve integrity, promoting epithelial healing, and modulating immune responses [122,123]. It plays a vital role in neutralizing reactive oxygen species (ROS), reducing chronic inflammation, and enhancing nerve regeneration by interacting with immune cells to facilitate tissue repair [124]. However, aging correlates with diminished SP levels, impairing nerve healing, sustaining inflammation, and increasing susceptibility to nerve damage. The resulting SP deficiency disrupts neuroimmune homeostasis, exacerbates oxidative stress, and promotes progressive corneal neurodegeneration, marking it as a significant contributor to age-associated corneal nerve dysfunction [124,125].

#### 5.1.3. Oxysterol Accumulation and Lipid Oxidation in Corneal and Neural Aging

Oxysterols, oxygenated derivatives of cholesterol, have emerged as key mediators linking lipid metabolism to age-related ocular diseases such as cataracts and age-related macular degeneration (AMD). In the human lens, high cholesterol levels and exposure to oxidative stressors promote the formation of oxysterols, including 7β-hydroxycholesterol, 7-ketocholesterol, and 25-hydroxycholesterol, which accumulate in cataractous lenses and are associated with disrupted cholesterol metabolism. In AMD, elevated oxysterols such as 24S-hydroxycholesterol, 27-hydroxycholesterol, and 7-ketocholesterol contribute to oxidative stress, mitochondrial dysfunction, inflammation, and neovascularization in retinal pigment epithelial cells [126]. Importantly, several oxysterols have been detected in the cornea, where they alter epithelial barrier integrity and keratocyte viability, suggesting a potential contribution to corneal aging and impaired wound healing [127]. Beyond the eye, oxysterols also influence neuronal excitability and synaptic transmission, modulating ion channels and neurotransmitter release through effects on membrane fluidity and NMDA receptor signaling [128]. Taken together, these findings indicate that oxysterol accumulation with age may contribute to corneal and neural dysfunction by amplifying oxidative and inflammatory cascades, an emerging area of interest that warrants deeper exploration in ocular neurobiology and aging research.

### 5.2. Cellular Energy Metabolism

Advancing age perturbs the metabolic axes that maintain mitochondrial bioenergetics in corneal nerves, most notably the insulin/IGF-1 (IIS) cascade, the mechanistic target of rapamycin (mTOR) network, and AMP-activated protein kinase (AMPK) signaling [34].

#### 5.2.1. Insulin/IGF-1 Signaling (IIS) Pathway

The insulin/IGF-1 signaling (IIS) pathway is a master regulator of neuronal metabolism, mitochondrial homeostasis, and stress resistance [34,129]. Chronic hyper-activation of IIS in late life reduces mitochondrial ATP production and up-regulates senescence-associated gene programs, thereby accelerating neurodegeneration, whereas partial IIS attenuation is consistently linked to lifespan extension across species [129,130,131]. In aged corneal nerves, ATP synthesis declines even though mitochondrial number rises, indicating bioenergetic inefficiency, an IIS-dependent defect that compromises axonal survival. Dysregulated IIS also resets key ageing genes: FOXO transcription factors, normally protective against oxidative stress, are suppressed, while mTOR activity is disinhibited, further driving protein accumulation and axonal loss [130]. Thus, tight modulation rather than wholesale inhibition of IIS is required to preserve corneal nerve integrity and delay age-related functional decline.

#### 5.2.2. mTOR Signaling Pathway

The mechanistic target of rapamycin (mTOR) coordinates growth-factor and nutrient cues to regulate protein synthesis, lipid metabolism, autophagy, and cytoskeletal dynamics through two biochemically distinct complexes, mTORC1 and mTORC2. Persistent mTORC1 hyper-activation suppresses autophagy, drives aberrant protein/lipid accumulation, and precipitates cellular dysfunction and neurodegeneration, including loss of corneal sensory axons. In contrast, mTORC2 supports actin remodelling, mitochondrial integrity, and pro-survival AKT signalling, thereby exerting a neuro-protective effect. Pharmacological or genetic attenuation of mTORC1 activity (e.g., with rapamycin or next-generation rapalogues) extends lifespan in multiple model organisms and improves neuronal longevity, highlighting selective mTORC1 inhibition, and maintenance of an appropriate mTORC1:mTORC2 balance as a promising therapeutic strategy for preserving corneal nerve integrity during ageing and disease [132,133,134].

#### 5.2.3. AMPK and Bioenergetic Homeostasis

AMP-activated protein kinase (AMPK) is the principal cellular energy sensor, activated by rises in the AMP/ATP ratio to restore metabolic balance. Once engaged, AMPK up-regulates catabolic pathways that generate ATP, enhances antioxidant defenses, and promotes autophagy-mediated clearance of damaged mitochondria, thereby curbing oxidative stress [135,136]. In corneal sensory axons, sustained AMPK activity preserves mitochondrial integrity, limits ROS build-up, and safeguards neuronal viability. Ageing blunts AMPK responsiveness: reduced kinase activity attenuates these protective programmes, exacerbates mitochondrial dysfunction, and increases susceptibility to apoptosis and axonal loss [136,137,138]. Pharmacological or dietary activation of AMPK using agents such as metformin, AICAR, or caloric-restriction mimetics thus represents a promising strategy to decelerate corneal nerve ageing and strengthen neuronal resilience [139].

### 5.3. Immunity and Corneal Nerve Aging

Ageing shifts the ocular surface toward a pro-inflammatory, neurodegenerative milieu and is associated with widespread immune dysregulation that profoundly affects corneal nerve homeostasis, regenerative capacity, and sensory function. Multiple neuroimmune factors including calcitonin gene-related peptide (CGRP), tau protein accumulation, chronic inflammation, and systemic immunosenescence contribute to age-associated degeneration of corneal nerves.

#### 5.3.1. Calcitonin Gene-Related Peptide (CGRP)

CGRP is a neuropeptide released from trigeminal sensory endings and plays a critical role in corneal nerve conduction, neuroprotection, immunomodulation, and epithelial integrity maintenance [140,141]. Notably, CGRP levels in human tears decline by approximately 8% annually with age, correlating strongly with reductions in subbasal nerve density [141]. The structural and functional integrity of corneal nerves is tightly linked to CGRP expression, and diminished CGRP levels disrupt neuroimmune signaling, compromise epithelial repair, and impair sensory feedback [142]. He et al. demonstrated that corneal nerve density in rats progressively declines with age, mirroring the loss of CGRP-positive subbasal nerve fibers underscoring CGRP’s neuroprotective role in the aging cornea [5]. The age-related depletion of CGRP may underlie observed reductions in corneal sensitivity, delayed wound healing, and heightened vulnerability to neuroimmune dysregulation.

#### 5.3.2. Tau Protein and Corneal Nerve Dysfunction

Tau protein, a microtubule-associated protein, is essential for axonal transport and mitochondrial trafficking along nerve fibers [143]. In aging populations, tau protein undergoes pathological hyperphosphorylation and aggregation, a hallmark of several neurodegenerative diseases, most notably Alzheimer’s disease. Recent evidence suggests that corneal nerve fibers may similarly accumulate hyperphosphorylated tau, leading to reductions in subbasal nerve fiber density and diminished regenerative potential. The pathogenic mechanisms underlying tau-related corneal nerve dysfunction likely involve mitochondrial impairment, axonal energy deficits, and dysregulated neuroimmune interactions. Due to the cornea’s unique accessibility and transparent anatomical structure, non-invasive in vivo corneal nerve imaging emerges as a promising diagnostic biomarker for early detection of tau-related neuropathology [144,145,146].

#### 5.3.3. Chronic Inflammation and Uric Acid

Although acute, low-grade inflammation can promote corneal nerve regeneration, persistent and excessive inflammatory responses ultimately compromise nerve integrity [81]. Aging is characterized by an increased propensity toward chronic systemic inflammation, termed “inflammaging,” partly driven by elevated uric acid levels commonly observed in older populations. Elevated uric acid exacerbates local cellular stress and amplifies inflammatory responses, contributing to corneal nerve degeneration and hindering regenerative processes [70]. Jia Y. et al. conducted a notable multicenter cross-sectional study involving over 143 healthy participants aged 21 to 80 years, utilizing in vivo confocal microscopy (IVCM) to assess corneal subbasal nerve parameters. This investigation demonstrated a significant, progressive decline in subbasal nerve density beginning around the fifth decade of life, with accelerated fiber loss noted beyond the age of 70 [64]. Moreover, the observed reduction in corneal sensitivity correlated directly with systemic inflammatory markers, including C-reactive protein, reinforcing the integrative connection between chronic low-grade inflammation and corneal nerve degeneration in older adults. These findings emphasize the critical need to evaluate systemic health influences, particularly inflammation and metabolic disruptions, when considering age-related corneal nerve changes [147].

#### 5.3.4. Immunosenescence and Corneal Nerve Vulnerability

With advancing age, the immune system undergoes immunosenescence, a multifaceted decline marked by skewed cytokine production (elevated IL-6, IL-8, TNF-α and diminished IL-10), sluggish phagocytosis and efferocytosis, impaired antigen presentation, and chronic, low-grade inflammasome activity. At the ocular surface these changes foster a persistent pro-inflammatory milieu: senescent immune and epithelial cells release a SASP rich in matrix-degrading enzymes and neuro-toxic cytokines, while age-related shifts toward Th17 dominance, weakened T-reg suppression, and constitutive NLRP3 activation sustain epithelial injury, slow axonal regrowth, and compromise debris clearance. The resulting failure to resolve inflammation accelerates corneal nerve attrition and delays wound healing after surgery, infection, or metabolic stress. Pre-clinical studies in aged mice demonstrate that dampening NLRP3 signaling, rejuvenating tissue-resident macrophages, or eliminating SASP-producing cells can hasten epithelial resurfacing and restore corneal nerve density, underscoring the therapeutic promise of targeting immune ageing to preserve ocular surface integrity [148,149].

In summary, age-related immune dysregulation significantly influences corneal nerve health through declining CGRP levels, pathological tau aggregation, chronic inflammation, and immunosenescence. Multi-modal approaches that replenish neurotrophic peptides, stabilise axonal transport, and recalibrate ocular surface immunity may together preserve corneal sensation and epithelial integrity in the elderly.

## 6. Therapeutic Approaches for Age-Related Corneal Nerve Degeneration

Ageing is associated with a progressive decline in corneal nerve integrity and a reduced intrinsic capacity for axonal repair, which collectively compromise the regenerative potential of the cornea. This deterioration is multifactorial, driven by chronic low-grade inflammation, increased oxidative stress, and a decline in neurotrophic support all of which disrupt the homeostatic neuro-immune axis essential for maintaining corneal health. Age-related neuro-immune dysregulation not only impairs the clearance of neurotoxic debris and resolution of inflammation but also alters the local microenvironment in a way that hinders nerve regeneration. Consequently, recovery following corneal injury or surgical intervention is markedly delayed in aged individuals, underscoring the importance of targeting these molecular and cellular deficits to restore corneal nerve function in the elderly. This section outlines current and emerging strategies including stem cell-based approaches, extracellular vesicle (EV) therapy, growth factor supplementation, neuroprotective compounds, biomaterial scaffolds, and preventive interventions that aim to restore corneal nerve structure and function in aging individuals. (Table 1)

### 6.1. Stem-Cell-Based Therapies

Stem cell therapies offer a promising strategy to replenish neurotrophic support and promote axonal regrowth in the aging cornea, where nerve density and regenerative capacity decline with age. Various stem cell types spanning adult tissue-derived cells to pluripotent stem cells have been explored for their potential to differentiate into peripheral neural phenotypes or to secrete neuroprotective factors that aid nerve regeneration:

#### 6.1.1. Corneal Stromal Stem Cells (CSSCs)

These are adult stem cells resident in the corneal stroma/limbus. CSSCs can be expanded in vitro for years without losing potency and have demonstrated multipotency, differentiating into keratocytes, osteocytes, and neuron-like cells [150]. Given their ease of autologous isolation from a small limbal biopsy and their neurogenic potential, CSSCs have been investigated as a therapy to restore corneal nerves and function [151]. Preclinical studies indicate that transplanting CSSCs into injured corneas can promote nerve regeneration, likely by both direct differentiation and release of trophic factors (e.g., nerve growth factor, NGF) [152].

#### 6.1.2. Bone Marrow Mesenchymal Stem Cells (BM-MSCs)

Adult stem cells, such as bone marrow-derived MSCs, possess the capacity to differentiate into mesodermal lineages, including fat, bone, muscle, and cartilage. Additionally, certain studies suggest that these cells may also differentiate into neuronal or glial lineages under specific in vitro conditions [153,154,155,156]. Yu et al. proposed a protocol for inducing BM-MSCs toward neuronal phenotypes, involving an initial treatment with Forskolin and fibroblast growth factor-2 (FGF2) for 24 h, followed by sonic hedgehog and retinoic acid administration. This approach successfully generated cells expressing glutamatergic neuronal markers (VGluT1, calretinin, P2X3) and neuronal proteins (NeuN, MAP-2, GluR4) [157]. BM-MSCs are well-known for secreting an array of growth factors and for supporting nerve repair [158]. They can even trans-differentiate towards Schwann cell-like and neuronal phenotypes in peripheral nerve environments [159,160]. BM-MSC effectively produce and secrete neurotrophins, such as nerve growth factor (NGF), brain-derived neurotrophic factor (BDNF), glial-cell-line-derived neurotrophic factor (GDNF), and ciliary neurotrophic factor (CNTF) [158].

#### 6.1.3. Adipose Tissue-Derived MSCs (ADSCs)

ADSCs exhibit greater yields, enhanced proliferation rates, and lower immunogenicity compared to bone marrow-derived MSCs [161]. Additionally, they improve the neural regeneration environment by reducing inflammation [162]. In vitro, ADSCs produce specific mRNAs promoting the release of neuroregenerative paracrine factors, including BDNF, glial-growth-like factor (GGF), neuregulin-1 (NRG-1), VEGF, hepatocyte growth factor (HGF), and insulin-like growth factor (IGF). Moreover, ADSCs have robust multilineage differentiation capabilities, notably toward Schwann-like cells, suggesting a promising therapeutic potential for peripheral nerve repair [162,163,164].

#### 6.1.4. Fetal Tissue-Derived MSCs

Umbilical cord (UC) and amniotic fluid represent primitive fetal sources of MSCs, accessible through noninvasive procedures with minimal genetic damage risk and strong proliferative capacities. UC-derived MSCs (UCMSCs), obtained from cord blood and Wharton’s jelly [165], offer significant advantages including easy accessibility, immunological inertness, lack of ethical issues, and a low risk of graft-vs-host disease [158]. Although rare reports mention tumorigenesis post-transplantation, UCMSCs produce neurotrophic factors such as GDNF, VEGF, CNF, NGF, and BDNF. Following specific induction treatments such as mercaptoethanol, retinoic acid, UCMSCs express Schwann cell markers and enhance nerve regeneration in animal models [166]. Wharton’s jelly-derived MSCs possess unique properties bridging embryonic and adult stem cells. They express mesenchymal lineage markers, can differentiate into Schwann cell-like cells, and secrete NGF, BDNF, and NT-3 to support neurite growth in vitro [167]. These cells have low immunogenicity, are negative for MHC class II, exhibit minimal MHC class I expression, and can be cryogenically stored post-birth. Amniotic MSCs (AMSCs), derived from avascular amniotic mesoderm, are relatively non-immunogenic and support nerve regeneration by secreting angiogenic factors like VEGF and expressing chemokines (CCR2, CCR3, CCR5), enhancing migration, engraftment, and endothelial differentiation. Under specific conditions, AMSCs can differentiate toward neural phenotypes in vitro. While embryonic stem cells offer robust differentiation potential, their use is limited by ethical concerns and sourcing challenges [168,169].

#### 6.1.5. Dental Pulp-Derived MSCs

Human dental pulp is an accessible source of stem cells obtained easily from routine wisdom tooth extractions. These dental pulp-derived mesenchymal stem cells (DPSCs) exhibit self-renewal, multilineage differentiation capabilities, and immunomodulatory effects, notably through the secretion of IL-8, IL-6, and TGF-β, contributing to tissue regeneration [170]. Under appropriate in vitro conditions, DPSCs differentiate toward neuronal and Schwann cell phenotypes, expressing markers such as GFAP, nestin, βIII-tubulin, NF-200, and MAP-2. Additionally, studies demonstrate that DPSCs can facilitate peripheral nerve regeneration when combined with conduits or electromagnetic stimulation [171]. Despite encouraging efficacy, cell-based therapies face translational hurdles, cellular heterogeneity, immune rejection, tumourigenicity, batch-to-batch variability, and manufacturing cost all limit widespread adoption. To circumvent the limitations of live-cell delivery, attention has shifted to the secretome particularly small extracellular vesicles (EVs/exosomes) as a therapeutic proxy.

### 6.2. Cell-Free Therapies: Exosomes and Neurotrophic Factors

To overcome the limitations of direct cell transplantation, researchers have turned to cell-secreted factors—including soluble growth factors and extracellular vesicles—as a therapy to rejuvenate corneal nerves. Aging is linked to dysregulation of intercellular signaling, including altered EV profiles [172], which may lead to reduced trophic support for nerves. Augmenting the ocular surface with exogenous neurotrophic factors or EVs can potentially restore a pro-regenerative microenvironment in the aged cornea.

#### 6.2.1. Neurotrophic Growth Factors

A straightforward approach is supplementation with key nerve growth factors that decline with age. Nerve Growth Factor (NGF), for instance, is essential for corneal nerve maintenance and regeneration, and its expression in the cornea drops in older individuals [64,173]. Topical NGF application has proven efficacy in patients—cenegermin, a recombinant human NGF eye drop, was the first FDA-approved treatment for neurotrophic keratitis and significantly improves corneal healing and sensation by stimulating nerve regrowth [174,175]. Other neurotrophins like BDNF and NT-3, as well as cytokines released from the corneal epithelium, are known to support nerve fiber survival [176]. However, delivering proteins alone can be limited by short half-life and poor tissue retention. This has led to interest in secretomes and vesicle-based delivery systems as more sustained, targeted means to provide a cocktail of regenerative signals.

#### 6.2.2. Extracellular Vesicles (Exosomes)

Cell-derived exosomes (30–150 nm vesicles) and other EVs carry a rich cargo of growth factors, cytokines, lipids, and microRNAs that can recapitulate many of the parent cell’s therapeutic effects [151,177]. EVs from MSCs, in particular, contain multiple neurotrophic factors and have shown remarkable ability to enhance neural repair [178,179,180]. In ocular models, EVs have demonstrated pro-regenerative effects on both injured retinal ganglion cells and corneal nerves. Notably, EVs derived from MSCs have promoted axon regeneration and functional recovery in animal models of optic nerve injury [181]. In the cornea, recent studies report that EVs isolated from bone marrow MSCs or corneal stromal stem cells can stimulate corneal nerve regrowth after injury. For example, EVs harvested from 3D-cultured MSCs (which have enhanced secretory profiles) significantly increased corneal nerve fiber regeneration and elongation in vivo compared to controls, highlighting the potency of the secretome approach in nerve repair. These vesicles likely exert their benefits by transferring neurotrophic microRNAs and proteins to resident corneal cells, thereby activating axon growth pathways and modulating inflammation. (Combining proteomic and in vitro) [182,183,184,185] Importantly, EV-based therapy is inherently cell-free, avoiding issues of immune rejection and tumorigenesis. Another practical advantage of exosomes is their mode of delivery. EVs can be formulated as eye drops for topical application, offering a non-invasive treatment that can cover the entire corneal surface. In murine models, topically applied MSC-derived exosomes were efficiently taken up by corneal cells and accelerated wound closure, indicating successful delivery and bioactivity in vivo [186]. This facile delivery method stands in contrast to cell transplants that may require surgery or injections. Early-stage research and translational studies are now exploring engineered exosomes or concentrated EV eye drops for corneal nerve regeneration in conditions like neurotrophic keratopathy. While still in preclinical phases, the results are promising exosome treatments have been shown to restore nerve density and sensitivity in animal models of corneal nerve damage. In summary, cell-free exosome therapy represents a cutting-edge extension of stem cell medicine, leveraging the regenerative payload of stem cells without transplanting live cells. As the cornea ages and its endogenous repair mechanisms wane, such therapies could provide the needed neurotrophic boost to maintain corneal innervation. Further research, including controlled preclinical studies and clinical trials, is underway to determine the optimal sources (e.g., corneal vs. bone marrow MSC-EVs) and dosing regimens for exosome-based treatments [151]. If successful, exosome eye drops enriched with neuroregenerative factors could become a convenient and safe therapeutic option to rejuvenate aged corneal nerves and improve ocular surface health in the elderly.

### 6.3. Neuroprotective Strategies

The aging cornea experiences chronic inflammation, oxidative stress, and metabolic decline. Together, these factors contribute to progressive corneal nerve degeneration. Neuroprotective strategies are therefore crucial for preserving neuronal integrity and preventing further degeneration in older individuals. One such strategy involves the use of polyunsaturated fatty acids (PUFAs) like docosahexaenoic acid (DHA). These lipids mitigate inflammatory damage and enhance neurotrophic signaling, thereby providing essential support to aging corneal nerves [187,188]. Notably, combining DHA with nerve growth factor (NGF) exerts synergistic effects that significantly enhance postoperative corneal nerve regeneration after surgeries such as photorefractive keratectomy (PRK) and laser-assisted in situ keratomileusis (LASIK). This combined therapeutic approach is especially relevant for older patients with pre-existing corneal nerve deficits, who may experience greater neurodegenerative impacts from these procedures [189]. Additionally, pigment epithelium-derived factor (PEDF) confers further neuroprotection by promoting axonal regeneration in the aged cornea [190].

### 6.4. Biomaterial Scaffolds

Biomaterial scaffolds have emerged as a promising approach to address age-impaired corneal nerve healing. These scaffolds which include natural tissue-derived matrices and engineered hydrogels can serve as delivery platforms for neurotrophic factors, growth factors, and stem cell-derived bioactive molecules that promote axon regeneration [191]. By providing a sustained, localized release of therapeutic agents, scaffolds create a microenvironment conducive to nerve regrowth even in an aged or compromised cornea. For instance, tissue-engineered implants can be loaded with recombinant human nerve growth factor (rhNGF) or other neurotrophic factors to continuously bathe regenerating nerves in supportive cues. In a recent proof-of-concept, decellularized human SMILE-derived lenticules (refractive surgery tissue discs) embedded with NGF-releasing microparticles achieved controlled NGF release for up to one month. [192] Such sustained delivery is expected to counteract the diminished endogenous healing signals in older corneas and thereby promote nerve repopulation and functional recovery. Moreover, scaffolds can be combined with cellular therapies: mesenchymal stem cells or their secreted exosomes can be incorporated to provide a cocktail of regenerative factors (including NGF, hepatocyte growth factor, and others) that synergistically encourage nerve repair [193]. Taken together, a biomaterial scaffold can act as both a structural support and a bioactive depot, overcoming age-related healing deficiencies by continuously supplying the molecules and cells needed for nerve regeneration. A wide variety of scaffold designs have been explored for corneal nerve regeneration. These include biological scaffolds (e.g., decellularized corneal tissues or donor-derived stromal lenticules), synthetic hydrogels (engineered polymers or recombinant collagens), as well as cellular (cell-seeded) and acellular tissue-engineered constructs. Other approaches involve synthetic hydrogel patches or films that can be applied to an injured cornea to guide nerve ingrowth. Regardless of the format, an effective scaffold must integrate with the host tissue, allow cell migration, and permit regenerating nerve fibers to penetrate and form connections. Among the most promising biomaterials for corneal nerve repair are collagen-based scaffolds, which mimic the corneal stroma’s primary component. Recombinant human collagen (RHC) scaffolds have shown excellent biocompatibility and can serve as off-the-shelf substitutes for donor corneas [192,194,195]. In a landmark Phase I clinical trial, Fagerholm et al. implanted biosynthetic collagen implants in patients as an alternative to traditional corneal graft. Over a 24-month follow-up, these collagen implants became stably integrated into the host cornea with minimal complications. The patients’ corneas re-epithelialized fully, and native stromal cells migrated into the collagen scaffold, populating it with new living tissue [196]. Remarkably, nerve regeneration was also observed: regenerating corneal nerves grew into the implants, and corneal touch sensitivity was restored to levels equal to or even greater than those achieved with conventional human donor transplants. This demonstrated that a bioengineered collagen matrix can support not only structural healing but also functional nerve recovery in vivo. Importantly, these outcomes were achieved without the need for long-term immunosuppression, highlighting the scaffold’s biocompatibility and low immunogenicity. Researchers have enhanced collagen scaffolds by introducing biochemical modifications, notably creating a collagen–phosphorylcholine (MPC) hydrogel scaffold. This hybrid scaffold, engineered by Liu et al., exhibits improved mechanical strength, optical clarity, and enzymatic stability, while preserving collagen’s biointeractive properties that support cell attachment and nerve fiber growth [195]. In an inflammation-challenged mini-pig model, collagen-MPC scaffolds not only suppressed scarring and neovascularization, but also enabled faster reinnervation and blink reflex recovery than their collagen-only counterparts [197]. Scaffold-based strategies effectively address age-related declines in corneal nerve regeneration by providing structural support and delivering regenerative signals. In elderly patients with impaired nerve healing, scaffolds can deliver sustained NGF release and cellular support, enhancing nerve regrowth and restoring corneal sensation. Early clinical results demonstrate successful nerve regeneration into collagen scaffolds, comparable to donor tissue. Future advancements like integrating specific neurotrophic factors, gene-activated matrices, or patient-derived stem cells could further enhance therapeutic efficacy. Overall, biomaterial scaffolds offer a transformative and lasting solution for corneal neuroregeneration in age-related neuropathies.

### 6.5. Proteoglycans and Inflammation Modulation

Aging increases neuroimmune dysregulation, leading to chronic low-grade inflammation that interferes with corneal nerve homeostasis. Studies indicate that aging impairs ocular surface homeostasis and leads to corneal nerve loss through two main mechanisms: (1) the accumulation of toxic, pro-inflammatory molecules with age that create a neurotoxic environment, and (2) a decline in the regenerative capacity of corneal nerves. This persistent inflammation (“inflammaging”) can hinder nerve maintenance and repair in older corneas [64]. Proteoglycans such as decorin—a small leucine-rich proteoglycan in the corneal extracellular matrix—play a key role in modulating this inflammatory response. Decorin has demonstrated potent immunomodulatory and neuroregenerative effects in the cornea. For example, in mouse models of corneal injury, topical decorin treatment significantly attenuated inflammation and enhanced nerve recovery: decorin-treated corneas showed fewer infiltrating neutrophils and macrophages and a higher density of regenerating corneal nerves compared to controls. At the same time, decorin promoted the recruitment or activation of intraepithelial dendritic cells (DCs) in the acute healing phase. These immunomodulatory actions—reducing pro-inflammatory macrophage/neutrophil activity while boosting DC presence—create a more regenerative environment and were associated with faster wound re-epithelialization and improved sensory nerve regeneration. Notably, the ability of decorin to suppress neutrophil influx was lost in DC-deficient mice, indicating that decorin engages DCs to orchestrate nerve repair [198]. This supports the hypothesis that decorin induces corneal nerve regeneration at least in part by activating dendritic cells, thereby enhancing the neuroimmune crosstalk favorable for nerve regrowth. Because of its dual anti-scarring and anti-inflammatory properties, decorin is being explored in biomaterial-based therapies for corneal nerve repair. One study demonstrated that a decorin-loaded fluid gel eye drop provided prolonged release to the cornea, which promoted scarless corneal wound healing and reduced opacity in a murine infection model [199]. Furthermore, topical decorin application in injured corneas has been shown to stimulate nerve regeneration even in challenging conditions (e.g., diabetic or aged corneas), offering a potential therapeutic avenue for aging eyes. Given that aged corneas are prone to persistent inflammation and fibrosis, targeting the inflammatory cascade with decorin or similar proteoglycans may confer long-term neuroprotection. In summary, proteoglycan-based interventions (such as decorin therapy) hold promise to modulate the inflammatory microenvironment of the aging cornea—enhancing dendritic cell activity, reducing macrophage-mediated damage, and ultimately promoting corneal nerve repair and regrowth [200].

### 6.6. Preventive Strategies to Slow Corneal Nerve Aging

Preventing further corneal nerve deterioration in older individuals is critical for preserving corneal sensation and delaying neurodegeneration. Key preventive strategies include:

#### 6.6.1. Avoiding Neurotoxic Preservatives (e.g., BAK)

Long-term use of benzalkonium chloride (BAK), a common preservative in eye drops, can exacerbate chronic inflammation and directly damage corneal nerves. BAK is well known to cause cytotoxic harm to the ocular surface. It induces corneal epithelial apoptosis, conjunctival inflammation, tear film instability, and other signs of ocular surface disease. Chronic exposure to BAK (for instance, in daily glaucoma medications) has been shown to lead to dry eye disease and neurosensory abnormalities, including ocular pain and reduced corneal sensitivity [96]. In animal models, topical BAK causes significant corneal nerve fiber loss and impaired wound healing, with dose- and time-dependent severity [201]. Many of these adverse effects may only partially recover after stopping BAK, especially if exposure was prolonged. Therefore, it is recommended to minimize or eliminate BAK use in older patients whenever possible. This can be achieved by switching to preservative-free formulations or using alternative, less-toxic preservatives.

#### 6.6.2. Preservative-Free Lubrication

Maintaining ocular surface homeostasis with preservative-free artificial tears is an important preventive measure, particularly for elderly individuals with a history of dry eye disease. Corneal nerves and tear film stability are interdependent; healthy nerves promote tearing and blinking reflexes, while a stable tear film protects the corneal epithelium and nerves. [148] Regular use of preservative-free lubricating eye drops (especially those containing tear components like sodium hyaluronate) can help break this vicious cycle. In addition to avoiding preservative toxicity, these drops improve hydration and reduce friction/inflammation on the cornea. Notably, sodium hyaluronate (HA) itself may have neuroprotective benefits: in a murine BAK-induced dry eye model, concurrent treatment with HA not only protected the epithelium but also promoted corneal nerve regeneration, restoring nerve density and corneal sensitivity to near-normal levels [202]. Such findings underscore that supporting the tear film with appropriate therapy can mitigate ongoing subclinical inflammation and foster an environment conducive to nerve fiber maintenance and repair.

#### 6.6.3. Topical Neuroprotective Vitamins (B_12_ and D)

Pharmacological interventions aimed at enhancing nerve health are emerging, with particular relevance to patients at risk for diabetic corneal neuropathy (a condition that disproportionately affects the aging population). Vitamin B_12_ (cobalamin) has shown neuroprotective and neurotrophic properties. In a clinical trial on diabetic patients, a topical combination of citicoline and vitamin B_12_ eye drops significantly improved corneal nerve morphology and sensitivity over 6–18 months compared to placebo, suggesting a genuine neuroregenerative effect [108]. This supports the use of B_12_ supplementation to help repair or prevent small-fiber corneal nerve damage in diabetes. Vitamin D is another crucial factor for nerve health. Older adults are often deficient in vitamin D, and studies have found that vitamin D deficiency correlates with worse peripheral neuropathy. In diabetics, low serum vitamin D levels are associated with more severe and painful neuropathy, and notably with lower corneal nerve fiber densities observed on corneal confocal microscopy [203]. Vitamin D appears to support corneal nerves by boosting neurotrophic factors and modulating immunity. In experimental models, topical active vitamin D_3_ analogs (such as 1,25-D_3_) markedly enhanced corneal nerve regeneration after injury in diabetic mice [204]. Therefore, ensuring adequate vitamin D status and potentially using topical vitamin D in research settings may provide neuroprotective benefits for the cornea. In summary, addressing modifiable nutritional deficits (like B_12_ and D) is a promising adjunct strategy to slow corneal nerve aging and protect against neuropathy in the elderly [205].

Aging corneal nerves undergo progressive structural and functional decline, resulting in reduced regenerative potential, increased vulnerability to damage, and impaired neuroimmune regulation. The strategies discussed here ranging from stem cell-derived therapies and growth factor interventions to scaffold-based neurotrophic delivery and preventive neuroprotection, offer a comprehensive framework for mitigating age-related corneal nerve degeneration. The integration of biomaterial engineering, exosome-based therapies, and targeted neurotrophic support represents a transformative shift in the management of aging-associated corneal neuropathies, paving the way for more effective and long-lasting therapeutic solutions.

## 7. Conclusions

Aging of the cornea leads to progressive structural and functional deterioration of corneal nerves. In healthy adults, corneal subbasal nerve density steadily declines with advancing age, even though certain morphological features—such as nerve tortuosity and branching patterns—may remain relatively unchanged in older individuals. Functionally, this neurodegeneration manifests as reduced corneal sensitivity, impaired wound healing, and diminished neural regenerative capacity, which collectively heighten the risk of ocular surface disorders (for example, persistent epithelial defects and neurotrophic keratitis) in the elderly. At the molecular level, multiple interrelated mechanisms drive age-related corneal nerve loss: chronic oxidative stress and mitochondrial dysfunction limit axonal energy supply; immunosenescence and low-grade inflammation create a hostile tissue microenvironment; and a decline in neurotrophic factors (such as nerve growth factor and substance P) undermines neuronal survival and repair. Moreover, these intrinsic aging processes are compounded by extrinsic risk factors common in older patients—including metabolic diseases (e.g., diabetes mellitus), cumulative corneal injuries from surgeries or infections, chronic ocular surface inflammation (dry eye disease), and long-term exposure to neurotoxic medications or preservatives—which further accelerate corneal nerve degeneration.

A multifaceted therapeutic approach has emerged to counteract these degenerative changes and preserve corneal innervation in aging eyes. Cell-based therapies, cell-free approaches, growth factor supplementation, neuroprotective compounds, and bioengineered biomaterial scaffolds effectively support nerve regrowth despite the diminished endogenous healing capacity of older corneas. Collectively, these interventions help maintain or restore corneal nerve density and function in the aging eye, translating to better corneal sensation and ocular surface health in older adults. Equally important are preventive measures to slow age-related neurodegeneration: avoiding chronic exposure to iatrogenic insults (such as long-term preservative-containing eye drops), controlling systemic conditions (like diabetes and ocular surface inflammation), and ensuring adequate nutritional support (particularly vitamins B_12_ and D) all contribute to preserving corneal innervation. In summary, addressing both the molecular drivers and the external risk factors of corneal nerve aging—through targeted therapies and risk-factor management—is essential for sustaining corneal nerve integrity and ocular surface homeostasis in the elderly.

## 8. Future Directions

Although significant advances have been made in elucidating the mechanisms of age-related corneal nerve degeneration, important gaps remain. Continued research is needed to: (1) define optimal cell-free therapies (e.g., extracellular vesicles) for aged corneal tissues, (2) explore senolytic strategies that target senescent Schwann cells and other glial subtypes, and (3) develop standardized imaging modalities to differentiate between physiological and pathological nerve changes in older individuals. Moreover, large-scale clinical trials are crucial to determine the long-term safety and efficacy of emerging treatments such as stem cell-derived EVs or biocompatible scaffolds to foster functional nerve regeneration and reduce the burden of age-related corneal neuropathies. By integrating these cutting-edge approaches, it may be possible to preserve or restore corneal nerve health well into advanced age.

## Figures and Tables

**Table 1:** Comparison of Neuroprotective Factors and Growth Factors in Corneal Nerve Health.

Category	Neuroprotective Factors	Growth Factors
Definition	Agents that protect existing neurons and promote overall nerve health by reducing damage and inflammation.	Biological molecules that stimulate cell growth, proliferation, differentiation, and regeneration.
Mechanism of Action	Primarily reduces oxidative stress, inflammation, and apoptosis, maintaining neuronal integrity and function.	Directly stimulates axonal outgrowth, nerve repair, and neuronal survival, actively regenerating damaged nerves.
Examples	- Omega-3 PUFAs (DHA, resolvins) reduce inflammation and enhance neurotrophic signaling. - PEDF enhances axonal growth and neuroprotection.	- Nerve Growth Factor (NGF) & rhNGF promote corneal nerve regeneration. - IGF-1 enhances mitochondrial activity and subbasal nerve density. - VEGF promotes corneal nerve regeneration.
Clinical Applications	Helps maintain corneal nerve function and prevents degeneration, especially in aging or post-surgical patients.	Used in therapeutic applications for neurodegenerative diseases like NK, glaucoma, and corneal injuries.
Post-Surgical Use	DHA + NGF therapy enhances post-PRK and LASIK nerve regeneration.	NGF, IGF-1, and PRP promote corneal nerve regeneration after nerve damage.

## Data Availability

Not applicable.

## Supplementary Materials

The following supporting information can be downloaded at: https://www.mdpi.com/article/10.3390/cells14211730/s1.

## References

[1] Arai H., Ouchi Y., Yokode M., Ito H., Uematsu H., Eto F., Oshima S., Ota K., Saito Y., Sasaki H. (2012). Toward the Realization of a Better Aged Society: Messages from Gerontology and Geriatrics. Geriatr. Gerontol. Int..

[2] Verdu E., Ceballos D., Vilches J.J., Navarro X. (2000). Influence of Aging on Peripheral Nerve Function and Regeneration. J. Peripher. Nerv. Syst..

[3] Rózsa A.J., Beuerman R.W. (1982). Density and Organization of Free Nerve Endings in the Corneal Epithelium of the Rabbit. Pain.

[4] Marfurt C.F., Cox J., Deek S., Dvorscak L. (2010). Anatomy of the Human Corneal Innervation. Exp. Eye Res..

[5] He J., Pham T.L., Bazan H.E.P. (2021). Neuroanatomy and Neurochemistry of Rat Cornea: Changes with Age. Ocul. Surf..

[6] He J., Bazan N.G., Bazan H.E.P. (2010). Mapping the Entire Human Corneal Nerve Architecture. Exp. Eye Res..

[7] Al-Aqaba M.A., Dhillon V.K., Mohammed I., Said D.G., Dua H.S. (2019). Corneal Nerves in Health and Disease. Prog. Retin. Eye Res..

[8] Gurnani B., Feroze K.B., Patel B.C. (2025). Neurotrophic Keratitis. StatPearls.

[9] Lafosse E., Wolffsohn J., Talens-Estarelles C., García-Lázaro S. (2020). Presbyopia and the Aging Eye: Existing Refractive Approaches and Their Potential Impact on Dry Eye Signs and Symptoms. Contact Lens Anterior Eye.

[10] Gipson I.K. (2013). Age-Related Changes and Diseases of the Ocular Surface and Cornea. Investig. Ophthalmol. Vis. Sci..

[11] Baratz K.H., Nau C.B., Winter E.J., McLaren J.W., Hodge D.O., Herman D.C., Bourne W.M. (2006). Effects of Glaucoma Medications on Corneal Endothelium, Keratocytes, and Subbasal Nerves Among Participants in the Ocular Hypertension Treatment Study. Cornea.

[12] Martone G., Frezzotti P., Tosi G.M., Traversi C., Mittica V., Malandrini A., Pichierri P., Balestrazzi A., Motolese P.A., Motolese I. (2009). An In Vivo Confocal Microscopy Analysis of Effects of Topical Antiglaucoma Therapy with Preservative on Corneal Innervation and Morphology. Am. J. Ophthalmol..

[13] Chang P.-Y., Carrel H., Huang J.-S., Wang I.-J., Hou Y.-C., Chen W.-L., Wang J.-Y., Hu F.-R. (2006). Decreased Density of Corneal Basal Epithelium and Subbasal Corneal Nerve Bundle Changes in Patients with Diabetic Retinopathy. Am. J. Ophthalmol..

[14] Del Castillo J.M.B., Wasfy M.A.S., Fernandez C., Garcia-Sanchez J. (2004). An In Vivo Confocal Masked Study on Corneal Epithelium and Subbasal Nerves in Patients with Dry Eye. Investig. Ophthalmol. Vis. Sci..

[15] Kitazawa K., Inomata T., Shih K., Hughes J.-W.B., Bozza N., Tomioka Y., Numa K., Yokoi N., Campisi J., Dana R. (2022). Impact of Aging on the Pathophysiology of Dry Eye Disease: A Systematic Review and Meta-Analysis. Ocul. Surf..

[16] Patel D.V., McGhee C.N.J. (2009). In Vivo Confocal Microscopy of Human Corneal Nerves in Health, in Ocular and Systemic Disease, and Following Corneal Surgery: A Review. Br. J. Ophthalmol..

[17] Kowtharapu B.S., Stachs O. (2020). Corneal Cells: Fine-Tuning Nerve Regeneration. Curr. Eye Res..

[18] Ogrodnik M., Salmonowicz H., Gladyshev V.N. (2019). Integrating Cellular Senescence with the Concept of Damage Accumulation in Aging: Relevance for Clearance of Senescent Cells. Aging Cell.

[19] López-Otín C., Blasco M.A., Partridge L., Serrano M., Kroemer G. (2023). Hallmarks of Aging: An Expanding Universe. Cell.

[20] Baechle J.J., Chen N., Makhijani P., Winer S., Furman D., Winer D.A. (2023). Chronic Inflammation and the Hallmarks of Aging. Mol. Metab..

[21] Ogrodnik M. (2021). Cellular Aging beyond Cellular Senescence: Markers of Senescence Prior to Cell Cycle Arrest in Vitro and in Vivo. Aging Cell.

[22] Ferrucci L., Gonzalez-Freire M., Fabbri E., Simonsick E., Tanaka T., Moore Z., Salimi S., Sierra F., De Cabo R. (2020). Measuring Biological Aging in Humans: A Quest. Aging Cell.

[23] Tripathi U., Misra A., Tchkonia T., Kirkland J.L. (2021). Impact of Senescent Cell Subtypes on Tissue Dysfunction and Repair: Importance and Research Questions. Mech. Ageing Dev..

[24] Li C., Zhou L., Sun H., Yang M. (2024). Age-Related Macular Degeneration: A Disease of Cellular Senescence and Dysregulated Immune Homeostasis. Clin. Interv. Aging.

[25] Zhang Y., Huang S., Xie B., Zhong Y. (2024). Aging, Cellular Senescence, and Glaucoma. Aging Dis..

[26] Sreekumar P.G., Hinton D.R., Kannan R. (2020). The Emerging Role of Senescence in Ocular Disease. Oxidative Med. Cell. Longev..

[27] Soleimani M., Cheraqpour K., Koganti R., Djalilian A.R. (2023). Cellular Senescence and Ophthalmic Diseases: Narrative Review. Graefe’s Arch. Clin. Exp. Ophthalmol..

[28] Ceballos D., Cuadras J., Verdú E., Navarro X. (1999). Morphometric and Ultrastructural Changes with Ageing in Mouse Peripheral Nerve. J. Anat..

[29] Knox C.A., Kokmen E., Dyck P.J. (1989). Morphometric Alteration of Rat Myelinated Fibers with Aging. J. Neuropathol. Exp. Neurol..

[30] Adinolfi A.M., Yamuy J., Morales F.R., Chase M.H. (1991). Segmental Demyelination in Peripheral Nerves of Old Cats. Neurobiol. Aging.

[31] Thomas P.K., King R.H.M., Sharma A.K. (1980). Changes with Age in the Peripheral Nerves of the Rat: An Ultrastructural Study. Acta Neuropathol..

[32] Scheibel M.E., Lindsay R.D., Tomiyasu U., Scheibel A.B. (1975). Progressive Dendritic Changes in Aging Human Cortex. Exp. Neurol..

[33] López-Teros M., Alarcón-Aguilar A., López-Diazguerrero N.E., Luna-López A., Königsberg M. (2022). Contribution of Senescent and Reactive Astrocytes on Central Nervous System Inflammaging. Biogerontology.

[34] Zhao J., Han Z., Ding L., Wang P., He X., Lin L. (2024). The Molecular Mechanism of Aging and the Role in Neurodegenerative Diseases. Heliyon.

[35] Pestronk A., Drachman D.B., Griffin J.W. (1980). Effects of Aging on Nerve Sprouting and Regeneration. Exp. Neurol..

[36] Choi S.-J., Harii K., Lee M.-J., Furuya F., Ueda K. (1995). Electrophysiological, Morphological, and Morphometric Effects of Aging on Nerve Regeneration in Rats. Scand. J. Plast. Reconstr. Surg. Hand Surg..

[37] Fuentes-Flores A., Geronimo-Olvera C., Girardi K., Necuñir-Ibarra D., Patel S.K., Bons J., Wright M.C., Geschwind D., Hoke A., Gomez-Sanchez J.A. (2023). Senescent Schwann Cells Induced by Aging and Chronic Denervation Impair Axonal Regeneration Following Peripheral Nerve Injury. EMBO Mol. Med..

[38] Hanani M., Spray D.C., Huang T.-Y. (2023). Age-Related Changes in Neurons and Satellite Glial Cells in Mouse Dorsal Root Ganglia. Int. J. Mol. Sci..

[39] Collins K.J., Exton-Smith A.N., James M.H., Oliver D.J. (1980). Functional Changes in Autonomic Nervous Responses with Ageing. Age Ageing.

[40] Budni J., Bellettini-Santos T., Mina F., Garcez M.L., Zugno A.I. (2015). The Involvement of BDNF, NGF and GDNF in Aging and Alzheimer’s Disease. Aging Dis..

[41] Yazdankhah M., Shang P., Ghosh S., Hose S., Liu H., Weiss J., Fitting C.S., Bhutto I.A., Zigler J.S., Qian J. (2021). Role of Glia in Optic Nerve. Prog. Retin. Eye Res..

[42] Mikelberg F.S., Drance S.M., Schulzer M., Yidegiligne H.M., Weis M.M. (1989). The Normal Human Optic Nerve. Ophthalmology.

[43] Jonas J.B., Schmidt A.M., Müller-Bergh J.A., Schlötzer-Schrehardt U.M., Naumann G.O. (1992). Human Optic Nerve Fiber Count and Optic Disc Size. Investig. Ophthalmol. Vis. Sci..

[44] Patel N.B., Lim M., Gajjar A., Evans K.B., Harwerth R.S. (2014). Age-Associated Changes in the Retinal Nerve Fiber Layer and Optic Nerve Head. Investig. Ophthalmol. Vis. Sci..

[45] Kanamori A., Escano M.F.T., Eno A., Nakamura M., Maeda H., Seya R., Ishibashi K., Negi A. (2003). Evaluation of the Effect of Aging on Retinal Nerve Fiber Layer Thickness Measured by Optical Coherence Tomography. Ophthalmologica.

[46] Celebi A.R.C., Mirza G.E. (2013). Age-Related Change in Retinal Nerve Fiber Layer Thickness Measured with Spectral Domain Optical Coherence Tomography. Investig. Ophthalmol. Vis. Sci..

[47] Budenz D.L., Anderson D.R., Varma R., Schuman J., Cantor L., Savell J., Greenfield D.S., Patella V.M., Quigley H.A., Tielsch J. (2007). Determinants of Normal Retinal Nerve Fiber Layer Thickness Measured by Stratus OCT. Ophthalmology.

[48] Alasil T., Wang K., Keane P.A., Lee H., Baniasadi N., De Boer J.F., Chen T.C. (2013). Analysis of Normal Retinal Nerve Fiber Layer Thickness by Age, Sex, and Race Using Spectral Domain Optical Coherence Tomography. J. Glaucoma.

[49] Harwerth R.S., Wheat J.L. (2008). Modeling the Effects of Aging on Retinal Ganglion Cell Density and Nerve Fiber Layer Thickness. Graefe’s Arch. Clin. Exp. Ophthalmol..

[50] Balazsi A.G., Rootman J., Drance S.M., Schulzer M., Douglas G.R. (1984). The Effect of Age on the Nerve Fiber Population of the Human Optic Nerve. Am. J. Ophthalmol..

[51] Zhi J.-J., Wu S.-L., Wu H.-Q., Ran Q., Gao X., Chen J.-F., Gu X.-M., Li T., Wang F., Xiao L. (2023). Insufficient Oligodendrocyte Turnover in Optic Nerve Contributes to Age-Related Axon Loss and Visual Deficits. J. Neurosci..

[52] Coleman-Belin J., Harris A., Chen B., Zhou J., Ciulla T., Verticchio A., Antman G., Chang M., Siesky B. (2023). Aging Effects on Optic Nerve Neurodegeneration. Int. J. Mol. Sci..

[53] Pahlaviani F.G., Yousefshahi F., Niktinat H., Roohipourmoallai R., Davoudi S., Iyer S.S.R., Bourbour S., Ebrahimiadib N. (2024). Local Anesthesia in 23-Gauge Vitreoretinal Surgery: A Comparison of Efficacy between Retrobulbar and Sub-Tenon’s Injection. Glob. Transl. Med..

[54] Niktinat H., Yousefshahi F., Fadakar K., Latifi G., Kalantaritarari F., Imani Fooladi M., Ghahari P., Goudarzi M., Ebrahimiadib N. (2024). Assessment of Ocular Neuropathic Pain Following Vitreoretinal Surgery Using 23-Gauge Sclerotomy. Glob. Transl. Med..

[55] Repka M.X., Quigley H.A. (1989). The Effect of Age on Normal Human Optic Nerve Fiver Number and Diameter. Ophthalmology.

[56] Moya F.J., Brigatti L., Caprioli J. (1999). Effect of Aging on Optic Nerve Appearance: A Longitudinal Study. Br. J. Ophthalmol..

[57] Wang C., Fu T., Xia C., Li Z. (2012). Changes in Mouse Corneal Epithelial Innervation with Age. Investig. Ophthalmol. Vis. Sci..

[58] De Silva M.E.H., Hill L.J., Downie L.E., Chinnery H.R. (2019). The Effects of Aging on Corneal and Ocular Surface Homeostasis in Mice. Investig. Ophthalmol. Vis. Sci..

[59] Stepp M.A., Pal-Ghosh S., Tadvalkar G., Williams A., Pflugfelder S.C., De Paiva C.S. (2018). Reduced Intraepithelial Corneal Nerve Density and Sensitivity Accompany Desiccating Stress and Aging in C57BL/6 Mice. Exp. Eye Res..

[60] Taurone S., Miglietta S., Spoletini M., Feher J., Artico M., Papa V., Matassa R., Familiari G., Gobbi P., Micera A. (2020). Age Related Changes Seen in Human Cornea in Formalin Fixed Sections and on Biomicroscopy in Living Subjects: A Comparison. Clin. Anat..

[61] Parissi M., Karanis G., Randjelovic S., Germundsson J., Poletti E., Ruggeri A., Utheim T.P., Lagali N. (2013). Standardized Baseline Human Corneal Subbasal Nerve Density for Clinical Investigations with Laser-Scanning in Vivo Confocal Microscopy. Investig. Ophthalmol. Vis. Sci..

[62] Roszkowska A.M., Wylęgała A., Gargano R., Spinella R., Inferrera L., Orzechowska-Wylęgała B., Aragona P. (2021). Impact of Corneal Parameters, Refractive Error and Age on Density and Morphology of the Subbasal Nerve Plexus Fibers in Healthy Adults. Sci. Rep..

[63] Zhao K., Yu H., Zheng X., Yang J., Wang X., Han Y., Jia L., Zhao J. (2021). Use of the Inferior Whorl for Detecting Age-Related Changes in Human Corneal Subbasal Nerve Plexus with Laser Scanning Confocal Microscopy. Ophthalmic Res..

[64] Chin J.Y., Liu C., Lee I.X.Y., Lin M.T.Y., Cheng C.-Y., Wong J.H.F., Teo C.L., Mehta J.S., Liu Y.-C. (2024). Impact of Age on the Characteristics of Corneal Nerves and Corneal Epithelial Cells in Healthy Adults. Cornea.

[65] Niederer R.L., Perumal D., Sherwin T., McGhee C.N.J. (2007). Age-Related Differences in the Normal Human Cornea: A Laser Scanning in Vivo Confocal Microscopy Study. Br. J. Ophthalmol..

[66] Grupcheva C.N., Wong T., Riley A.F., McGhee C.N. (2002). Assessing the Sub-basal Nerve Plexus of the Living Healthy Human Cornea by in Vivo Confocal Microscopy. Clin. Exp. Ophthalmol..

[67] Batawi H., Shalabi N., Joag M., Koru-Sengul T., Rodriguez J., Green P.T., Campigotto M., Karp C.L., Galor A. (2018). Sub-Basal Corneal Nerve Plexus Analysis Using a New Software Technology. Eye Contact Lens Sci. Clin. Pract..

[68] Erie J.C., McLaren J.W., Hodge D.O., Bourne W.M. (2005). The Effect of Age on the Corneal Subbasal Nerve Plexus. Cornea.

[69] Chao C., Wang R., Jones M., Karson N., Jussel A., Smith J., Richdale K., Harrison W. (2020). The Relationship Between Corneal Nerve Density and Hemoglobin A1c in Patients with Prediabetes and Type 2 Diabetes. Investig. Ophthalmol. Vis. Sci..

[70] Li M., Liu L., Shi Y., Sun L., Ma X., Zou J. (2021). Age-Related Differences in Corneal Nerve Regeneration after SMILE and the Mechanism Revealed by Metabolomics. Exp. Eye Res..

[71] Tavakoli M., Ferdousi M., Petropoulos I.N., Morris J., Pritchard N., Zhivov A., Ziegler D., Pacaud D., Romanchuk K., Perkins B.A. (2015). Normative Values for Corneal Nerve Morphology Assessed Using Corneal Confocal Microscopy: A Multinational Normative Data Set. Diabetes Care.

[72] Roszkowska A.M., Colosi P., Ferreri F.M.B., Galasso S. (2004). Age-Related Modifications of Corneal Sensitivity. Ophthalmologica.

[73] Alcalde I., Íñigo-Portugués A., González-González O., Almaraz L., Artime E., Morenilla-Palao C., Gallar J., Viana F., Merayo-Lloves J., Belmonte C. (2018). Morphological and Functional Changes in TRPM8-expressing Corneal Cold Thermoreceptor Neurons during Aging and Their Impact on Tearing in Mice. J. Comp. Neurol..

[74] Courson J.A., Rumbaut R.E., Burns A.R. (2024). Impact of Obesity and Age on Mouse Corneal Innervation at the Epithelial-Stromal Interface. Investig. Ophthalmol. Vis. Sci..

[75] Hillenaar T., Van Cleynenbreugel H., Remeijer L. (2012). How Normal Is the Transparent Cornea? Effects of Aging on Corneal Morphology. Ophthalmology.

[76] Badian R.A., Andréasson M., Svenningsson P., Utheim T.P., Lagali N. (2021). The Pattern of the Inferocentral Whorl Region of the Corneal Subbasal Nerve Plexus Is Altered with Age. Ocul. Surf..

[77] Chirapapaisan C., Thongsuwan S., Chirapapaisan N., Chonpimai P., Veeraburinon A. (2021). Characteristics of Corneal Subbasal Nerves in Different Age Groups: An in Vivo Confocal Microscopic Analysis. Clin. Ophthalmol..

[78] Patel D.V., Tavakoli M., Craig J.P., Efron N., McGhee C.N.J. (2009). Corneal Sensitivity and Slit Scanning In Vivo Confocal Microscopy of the Subbasal Nerve Plexus of the Normal Central and Peripheral Human Cornea. Cornea.

[79] Gambato C., Longhin E., Catania A.G., Lazzarini D., Parrozzani R., Midena E. (2015). Aging and Corneal Layers: An in Vivo Corneal Confocal Microscopy Study. Graefe’s Arch. Clin. Exp. Ophthalmol..

[80] Yang A.Y., Chow J., Liu J. (2018). Corneal Innervation and Sensation: The Eye and Beyond. Yale J. Biol. Med..

[81] Shaheen B.S., Bakir M., Jain S. (2014). Corneal Nerves in Health and Disease. Surv. Ophthalmol..

[82] Erie J.C., McLaren J.W., Hodge D.O., Bourne W.M. (2005). Recovery of Corneal Subbasal Nerve Density After PRK and LASIK. Am. J. Ophthalmol..

[83] Garcia-Gonzalez M., Cañadas P., Gros-Otero J., Rodriguez-Perez I., Cañones-Zafra R., Kozobolis V., Teus M.A. (2019). Long-Term Corneal Subbasal Nerve Plexus Regeneration after Laser in Situ Keratomileusis. J. Cataract. Refract. Surg..

[84] Denoyer A., Landman E., Trinh L., Faure J.-F., Auclin F., Baudouin C. (2015). Dry Eye Disease after Refractive Surgery. Ophthalmology.

[85] Liu Y.-C., Jung A.S.J., Chin J.Y., Yang L.W.Y., Mehta J.S. (2020). Cross-Sectional Study on Corneal Denervation in Contralateral Eyes Following SMILE Versus LASIK. J. Refract. Surg..

[86] Alam U., Jeziorska M., Petropoulos I.N., Asghar O., Fadavi H., Ponirakis G., Marshall A., Tavakoli M., Boulton A.J.M., Efron N. (2017). Diagnostic Utility of Corneal Confocal Microscopy and Intra-Epidermal Nerve Fibre Density in Diabetic Neuropathy. PLoS ONE.

[87] Kallinikos P., Berhanu M., O’Donnell C., Boulton A.J.M., Efron N., Malik R.A. (2004). Corneal Nerve Tortuosity in Diabetic Patients with Neuropathy. Investig. Ophthalmol. Vis. Sci..

[88] D’Onofrio L., Ferdousi M., Kalteniece A., Iqbal Z., Petropoulos I.N., Ponirakis G., Buzzetti R., Malik R.A., Soran H. (2022). Corneal Confocal Microscopy Identifies Small Nerve Fibre Damage in Patients with Hypertriglyceridemia. J. Clin. Lipidol..

[89] Teo C.H.Y., Liu C., Yu M., Lee I.X.Y., Anam A., Cheng C.-Y., Htunwai Y., Koh J.S., Chandran S.R., Liu Y.-C. (2025). Obesity Negatively Impacts Corneal Nerves in Patients with Diabetes Mellitus. Eye Vis..

[90] Mok E., Kam K.W., Young A.L. (2023). Corneal Nerve Changes in Herpes Zoster Ophthalmicus: A Prospective Longitudinal in Vivo Confocal Microscopy Study. Eye.

[91] Hamrah P., Cruzat A., Dastjerdi M.H., Prüss H., Zheng L., Shahatit B.M., Bayhan H.A., Dana R., Pavan-Langston D. (2013). Unilateral Herpes Zoster Ophthalmicus Results in Bilateral Corneal Nerve Alteration. Ophthalmology.

[92] Moein H.-R., Kheirkhah A., Muller R.T., Cruzat A.C., Pavan-Langston D., Hamrah P. (2018). Corneal Nerve Regeneration after Herpes Simplex Keratitis: A Longitudinal in Vivo Confocal Microscopy Study. Ocul. Surf..

[93] Luzu J., Labbé A., Réaux-Le Goazigo A., Rabut G., Liang H., Dupas B., Blautain B., Sène D., Baudouin C. (2022). In Vivo Confocal Microscopic Study of Corneal Innervation in Sjögren’s Syndrome with or without Small Fiber Neuropathy. Ocul. Surf..

[94] Bitirgen G., Kucuk A., Ergun M.C., Satirtav G., Malik R.A. (2023). Corneal Nerve Loss and Increased Langerhans Cells Are Associated with Disease Severity in Patients with Rheumatoid Arthritis. Eye.

[95] Bitirgen G., Kucuk A., Ergun M.C., Baloglu R., Gharib M.H., Al Emadi S., Ponirakis G., Malik R.A. (2021). Subclinical Corneal Nerve Fiber Damage and Immune Cell Activation in Systemic Lupus Erythematosus: A Corneal Confocal Microscopy Study. Transl. Vis. Sci. Technol..

[96] Sarkar J., Chaudhary S., Namavari A., Ozturk O., Chang J.-H., Yco L., Sonawane S., Khanolkar V., Hallak J., Jain S. (2012). Corneal Neurotoxicity Due to Topical Benzalkonium Chloride. Investig. Ophthalmol. Vis. Sci..

[97] Zhu X.-Y., Ge Q.-S., Li Z.-Y., Zhou L.-F., Bu Q.-W., Su Y., Wang X.-J., Zhou Q.-J., Pan X.-J., Hu D. (2024). Corneal Nerve Changes by Anti-Glaucoma Medications Examined by in Vivo Confocal Microscopy. Int. J. Ophthalmol..

[98] Tyler E.F., McGhee C.N.J., Lawrence B., Braatvedt G.D., Mankowski J.L., Oakley J.D., Sethi S., Misra S.L. (2022). Corneal Nerve Changes Observed by In Vivo Confocal Microscopy in Patients Receiving Oxaliplatin for Colorectal Cancer: The COCO Study. J. Clin. Med..

[99] Ferdousi M., Azmi S., Petropoulos I.N., Fadavi H., Ponirakis G., Marshall A., Tavakoli M., Malik I., Mansoor W., Malik R.A. (2015). Corneal Confocal Microscopy Detects Small Fibre Neuropathy in Patients with Upper Gastrointestinal Cancer and Nerve Regeneration in Chemotherapy Induced Peripheral Neuropathy. PLoS ONE.

[100] Jones M.R., Urits I., Wolf J., Corrigan D., Colburn L., Peterson E., Williamson A., Viswanath O. (2020). Drug-Induced Peripheral Neuropathy: A Narrative Review. Curr. Clin. Pharmacol..

[101] Petropoulos I.N., Bitirgen G., Ferdousi M., Kalteniece A., Azmi S., D’Onofrio L., Lim S.H., Ponirakis G., Khan A., Gad H. (2021). Corneal Confocal Microscopy to Image Small Nerve Fiber Degeneration: Ophthalmology Meets Neurology. Front. Pain Res..

[102] Dehghani C., Frost S., Jayasena R., Masters C.L., Kanagasingam Y. (2018). Ocular Biomarkers of Alzheimer’s Disease: The Role of Anterior Eye and Potential Future Directions. Investig. Ophthalmol. Vis. Sci..

[103] Ferrari G., Grisan E., Scarpa F., Fazio R., Comola M., Quattrini A., Comi G., Rama P., Riva N. (2014). Corneal Confocal Microscopy Reveals Trigeminal Small Sensory Fiber Neuropathy in Amyotrophic Lateral Sclerosis. Front. Aging Neurosci..

[104] Walsh J., Palandra J., Goihberg E., Deng S., Hurst S., Neubert H. (2023). Analysis of β-Nerve Growth Factor and Its Precursor during Human Pregnancy by Immunoaffinity-Liquid Chromatography Tandem Mass Spectrometry. Sci. Rep..

[105] Dinn R.B., Harris A., Marcus P.S. (2003). Ocular Changes in Pregnancy. Obstet. Gynecol. Surv..

[106] Ren X., Chou Y., Wang Y., Jing D., Chen Y., Li X. (2022). The Utility of Oral Vitamin B1 and Mecobalamin to Improve Corneal Nerves in Dry Eye Disease: An In Vivo Confocal Microscopy Study. Nutrients.

[107] Zhang Y., Fan D., Zhang Y., Zhang S., Wang H., Liu Z., Wang H. (2021). Using Corneal Confocal Microscopy to Compare Mecobalamin Intramuscular Injections vs Oral Tablets in Treating Diabetic Peripheral Neuropathy: A RCT. Sci. Rep..

[108] Fogagnolo P., Melardi E., Tranchina L., Rossetti L. (2020). Topical Citicoline and Vitamin B12 versus Placebo in the Treatment of Diabetes-Related Corneal Nerve Damage: A Randomized Double-Blind Controlled Trial. BMC Ophthalmol..

[109] Lu X., Vick S., Chen Z., Chen J., Watsky M.A. (2020). Effects of Vitamin D Receptor Knockout and Vitamin D Deficiency on Corneal Epithelial Wound Healing and Nerve Density in Diabetic Mice. Diabetes.

[110] Britten-Jones A.C., Craig J.P., Downie L.E. (2023). Omega-3 Polyunsaturated Fatty Acids and Corneal Nerve Health: Current Evidence and Future Directions. Ocul. Surf..

[111] Pang K., Lennikov A., Yang M. (2021). Hypoxia Adaptation in the Cornea: Current Animal Models and Underlying Mechanisms. Anim. Models Exp. Med..

[112] Chiang J.C.B., Tajbakhsh Z., Wolffsohn J.S. (2025). The Clinical Impact of Contact Lens Wear on Neural Structure and Function of the Cornea. Clin. Exp. Optom..

[113] Dikmetas O., Kocabeyoglu S., Mocan M.C. (2021). The Association between Meibomian Gland Atrophy and Corneal Subbasal Nerve Loss in Patients with Chronic Ocular Graft-versus-Host Disease. Curr. Eye Res..

[114] Kheirkhah A., Dohlman T.H., Amparo F., Arnoldner M.A., Jamali A., Hamrah P., Dana R. (2015). Effects of Corneal Nerve Density on the Response to Treatment in Dry Eye Disease. Ophthalmology.

[115] Harris D.L., Yamaguchi T., Hamrah P. (2018). A Novel Murine Model of Radiation Keratopathy. Investig. Ophthalmol. Vis. Sci..

[116] Abedi F., Hamrah P. (2018). Corneal Subbasal Nerve Recovery in an Acute Case of Ultraviolet Keratitis Treated with Autologous Serum Eye Drops. J. Ophthalmol..

[117] Soleimani M., Baharnoori S.M., Massoumi H., Cheraqpour K., Asadigandomani H., Mirzaei A., Ashraf M.J., Koganti R., Chaudhuri M., Ghassemi M. (2025). A Deep Dive into Radiation Keratopathy; Going beyond the Current Frontierss. Exp. Eye Res..

[118] Hoeijmakers J.H.J. (2009). DNA Damage, Aging, and Cancer. N. Engl. J. Med..

[119] Böhm E.W., Buonfiglio F., Voigt A.M., Bachmann P., Safi T., Pfeiffer N., Gericke A. (2023). Oxidative Stress in the Eye and Its Role in the Pathophysiology of Ocular Diseases. Redox Biol..

[120] Di G., Qi X., Zhao X., Zhang S., Danielson P., Zhou Q. (2017). Corneal Epithelium-Derived Neurotrophic Factors Promote Nerve Regeneration. Investig. Ophthalmol. Vis. Sci..

[121] Kropf E., Fahnestock M. (2021). Effects of Reactive Oxygen and Nitrogen Species on TrkA Expression and Signalling: Implications for proNGF in Aging and Alzheimer’s Disease. Cells.

[122] Singh R.B., Naderi A., Cho W., Ortiz G., Musayeva A., Dohlman T.H., Chen Y., Ferrari G., Dana R. (2022). Modulating the Tachykinin: Role of Substance P and Neurokinin Receptor Expression in Ocular Surface Disorders. Ocul. Surf..

[123] Tran M.T., Lausch R.N., Oakes J.E. (2000). Substance P Differentially Stimulates IL-8 Synthesis in Human Corneal Epithelial Cells. Investig. Ophthalmol. Vis. Sci..

[124] Suvas S. (2017). Role of Substance P Neuropeptide in Inflammation, Wound Healing, and Tissue Homeostasis. J. Immunol..

[125] Bonini S., Rama P., Olzi D., Lambiase A. (2003). Neurotrophic Keratitis. Eye.

[126] Olivier E., Rat P., Lizard G. (2024). Role of Oxysterols in Ocular Degeneration Mechanisms and Involvement of P2X7 Receptor. Implication of Oxysterols and Phytosterols in Aging and Human Diseases.

[127] Liu H., Gambino F., Algenio C.S., Wu C., Gao Y., Bouchard C.S., Qiao L., Bu P., Zhao S. (2020). Inflammation and Oxidative Stress Induced by Lipid Peroxidation Metabolite 4-Hydroxynonenal in Human Corneal Epithelial Cells. Graefe’s Arch. Clin. Exp. Ophthalmol..

[128] Linsenbardt A.J., Taylor A., Emnett C.M., Doherty J.J., Krishnan K., Covey D.F., Paul S.M., Zorumski C.F., Mennerick S. (2014). Different Oxysterols Have Opposing Actions at N-Methyl-d-Aspartate Receptors. Neuropharmacology.

[129] Sun Q., Li J., Gao F. (2014). New Insights into Insulin: The Anti-Inflammatory Effect and Its Clinical Relevance. World J. Diabetes.

[130] Johnson S.C., Rabinovitch P.S., Kaeberlein M. (2013). mTOR Is a Key Modulator of Ageing and Age-Related Disease. Nature.

[131] Bartke A., Sun L.Y., Longo V. (2013). Somatotropic Signaling: Trade-Offs Between Growth, Reproductive Development, and Longevity. Physiol. Rev..

[132] Liu G.Y., Sabatini D.M. (2020). mTOR at the Nexus of Nutrition, Growth, Ageing and Disease. Nat. Rev. Mol. Cell Biol..

[133] Porstmann T., Santos C.R., Griffiths B., Cully M., Wu M., Leevers S., Griffiths J.R., Chung Y.-L., Schulze A. (2008). SREBP Activity Is Regulated by mTORC1 and Contributes to Akt-Dependent Cell Growth. Cell Metab..

[134] Stallone G., Infante B., Prisciandaro C., Grandaliano G. (2019). mTOR and Aging: An Old Fashioned Dress. Int. J. Mol. Sci..

[135] Salminen A., Kaarniranta K., Kauppinen A. (2016). Age-Related Changes in AMPK Activation: Role for AMPK Phosphatases and Inhibitory Phosphorylation by Upstream Signaling Pathways. Ageing Res. Rev..

[136] Hardie D.G., Ross F.A., Hawley S.A. (2012). AMPK: A Nutrient and Energy Sensor That Maintains Energy Homeostasis. Nat. Rev. Mol. Cell Biol..

[137] Salminen A., Hyttinen J.M.T., Kaarniranta K. (2011). AMP-Activated Protein Kinase Inhibits NF-κB Signaling and Inflammation: Impact on Healthspan and Lifespan. J. Mol. Med..

[138] Sharma C., Kim S., Nam Y., Jung U.J., Kim S.R. (2021). Mitochondrial Dysfunction as a Driver of Cognitive Impairment in Alzheimer’s Disease. Int. J. Mol. Sci..

[139] Shukal D.K., Malaviya P.B., Sharma T. (2022). Role of the AMPK Signalling Pathway in the Aetiopathogenesis of Ocular Diseases. Hum. Exp. Toxicol..

[140] Hong X., Ding F., Xiong J., Wu Y., Chen W. (2023). Calcitonin Gene-Related Peptide and Persistent Corneal Pain: A Trigeminal Nerve Sensitization Perspective. Brain-X.

[141] Tummanapalli S.S., Willcox M.D.P., Issar T., Kwai N., Poynten A.M., Krishnan A.V., Pisarcikova J., Markoulli M. (2020). The Effect of Age, Gender and Body Mass Index on Tear Film Neuromediators and Corneal Nerves. Curr. Eye Res..

[142] Golebiowski B., Chao C., Stapleton F., Jalbert I. (2017). Corneal Nerve Morphology, Sensitivity, and Tear Neuropeptides in Contact Lens Wear. Optom. Vis. Sci..

[143] Vasic V., Barth K., Schmidt M.H.H. (2019). Neurodegeneration and Neuro-Regeneration—Alzheimer’s Disease and Stem Cell Therapy. Int. J. Mol. Sci..

[144] Li S., Shi S., Luo B., Xia F., Ha Y., Merkley K.H., Motamedi M., Zhang W., Liu H. (2022). Tauopathy Induces Degeneration and Impairs Regeneration of Sensory Nerves in the Cornea. Exp. Eye Res..

[145] Misra S.L., Kersten H.M., Roxburgh R.H., Danesh-Meyer H.V., McGhee C.N.J. (2017). Corneal Nerve Microstructure in Parkinson’s Disease. J. Clin. Neurosci..

[146] Jiao H., Downie L.E., Huang X., Wu M., Oberrauch S., Keenan R.J., Jacobson L.H., Chinnery H.R. (2020). Novel Alterations in Corneal Neuroimmune Phenotypes in Mice with Central Nervous System Tauopathy. J. Neuroinflamm..

[147] Khan A., Parray A., Akhtar N., Agouni A., Kamran S., Pananchikkal S.V., Priyanka R., Gad H., Ponirakis G., Petropoulos I.N. (2022). Corneal Nerve Loss in Patients with TIA and Acute Ischemic Stroke in Relation to Circulating Markers of Inflammation and Vascular Integrity. Sci. Rep..

[148] Galletti J.G., de Paiva C.S. (2021). The Ocular Surface Immune System through the Eyes of Aging. Ocul. Surf..

[149] Bian F., Xiao Y., Zaheer M., Volpe E.A., Pflugfelder S.C., Li D.-Q., de Paiva C.S. (2017). Inhibition of NLRP3 Inflammasome Pathway by Butyrate Improves Corneal Wound Healing in Corneal Alkali Burn. Int. J. Mol. Sci..

[150] Kumar A., Xu Y., Yang E., Du Y. (2018). Stemness and Regenerative Potential of Corneal Stromal Stem Cells and Their Secretome After Long-Term Storage: Implications for Ocular Regeneration. Investig. Ophthalmol. Vis. Sci..

[151] Kumar A., Yun H., Funderburgh M.L., Du Y. (2022). Regenerative Therapy for the Cornea. Prog. Retin. Eye Res..

[152] Nurković J.S., Vojinović R., Dolićanin Z. (2020). Corneal Stem Cells as a Source of Regenerative Cell-Based Therapy. Stem Cells Int..

[153] Lavorato A., Raimondo S., Boido M., Muratori L., Durante G., Cofano F., Vincitorio F., Petrone S., Titolo P., Tartara F. (2021). Mesenchymal Stem Cell Treatment Perspectives in Peripheral Nerve Regeneration: Systematic Review. Int. J. Mol. Sci..

[154] Jones S., Eisenberg H.M., Jia X. (2016). Advances and Future Applications of Augmented Peripheral Nerve Regeneration. Int. J. Mol. Sci..

[155] Karaca E.E., Soleimani M., Baharnoori M., Arabpour Z., Ashraf M.J., Jalilian E., Djalilian A.R. (2024). Protective Effects of Intracameral Mesenchymal Stem/Stromal Cells on Corneal Endothelial Injury: Exploring New Avenues in Regenerative Medicine. Investig. Ophthalmol. Vis. Sci..

[156] Kufta A., Soleimani M., Cheraqpour K., Baharnoori S.M., Massoumi H., Ashraf M.J., Koganti R., Ghassemi M., Anwar K.N., Jalilian E. (2024). The Role of Mesenchymal Stem/Stromal Cells in Corneal Wound Healing: A Comparative Study of Different Sources and Delivery Routes. Investig. Ophthalmol. Vis. Sci..

[157] Yu Z., Xu N., Zhang N., Xiong Y., Wang Z., Liang S., Zhao D., Huang F., Zhang C. (2019). Repair of Peripheral Nerve Sensory Impairments via the Transplantation of Bone Marrow Neural Tissue-Committed Stem Cell-Derived Sensory Neurons. Cell. Mol. Neurobiol..

[158] Kubiak C.A., Grochmal J., Kung T.A., Cederna P.S., Midha R., Kemp S.W.P. (2020). Stem-Cell-Based Therapies to Enhance Peripheral Nerve Regeneration. Muscle Nerve.

[159] Wang J., Ding F., Gu Y., Liu J., Gu X. (2009). Bone Marrow Mesenchymal Stem Cells Promote Cell Proliferation and Neurotrophic Function of Schwann Cells in Vitro and in Vivo. Brain Res..

[160] Zou X.-F., Zhang B.-Z., Qian W.-W., Cheng F.M. (2024). Bone Marrow Mesenchymal Stem Cells in Treatment of Peripheral Nerve Injury. World J. Stem Cells.

[161] Tong X. (2011). Transplantation of Adipose-Derived Stem Cells for Peripheral Nerve Repair. Int. J. Mol. Med..

[162] Widgerow A.D., Salibian A.A., Lalezari S., Evans G.R.D. (2013). Neuromodulatory Nerve Regeneration: Adipose Tissue-Derived Stem Cells and Neurotrophic Mediation in Peripheral Nerve Regeneration. J. Neurosci. Res..

[163] Wang Y., Guo Y., Wang D., Liu J., Pan J. (2019). Adipose Stem Cell-Based Clinical Strategy for Neural Regeneration: A Review of Current Opinion. Stem Cells Int..

[164] Kingham P.J., Kalbermatten D.F., Mahay D., Armstrong S.J., Wiberg M., Terenghi G. (2007). Adipose-Derived Stem Cells Differentiate into a Schwann Cell Phenotype and Promote Neurite Outgrowth in Vitro. Exp. Neurol..

[165] Drela K., Lech W., Figiel-Dabrowska A., Zychowicz M., Mikula M., Sarnowska A., Domanska-Janik K. (2016). Enhanced Neuro-Therapeutic Potential of Wharton’s Jelly–Derived Mesenchymal Stem Cells in Comparison with Bone Marrow Mesenchymal Stem Cells Culture. Cytotherapy.

[166] Matsuse D., Kitada M., Kohama M., Nishikawa K., Makinoshima H., Wakao S., Fujiyoshi Y., Heike T., Nakahata T., Akutsu H. (2010). Human Umbilical Cord-Derived Mesenchymal Stromal Cells Differentiate Into Functional Schwann Cells That Sustain Peripheral Nerve Regeneration. J. Neuropathol. Exp. Neurol..

[167] Shalaby S.M., El-Shal A.S., Ahmed F.E., Shaban S.F., Wahdan R.A., Kandel W.A., Senger M.S. (2017). Combined Wharton’s Jelly Derived Mesenchymal Stem Cells and Nerve Guidance Conduit: A Potential Promising Therapy for Peripheral Nerve Injuries. Int. J. Biochem. Cell Biol..

[168] Qiu C., Ge Z., Cui W., Yu L., Li J. (2020). Human Amniotic Epithelial Stem Cells: A Promising Seed Cell for Clinical Applications. Int. J. Mol. Sci..

[169] Li Y., Guo L., Ahn H.S., Kim M.H., Kim S. (2014). Amniotic Mesenchymal Stem Cells Display Neurovascular Tropism and Aid in the Recovery of Injured Peripheral Nerves. J. Cell. Mol. Med..

[170] Pisciotta A., Bertoni L., Vallarola A., Bertani G., Mecugni D., Carnevale G. (2020). Neural Crest Derived Stem Cells from Dental Pulp and Tooth-Associated Stem Cells for Peripheral Nerve Regeneration. Neural Regen. Res..

[171] Luo L., He Y., Wang X., Key B., Lee B.H., Li H., Ye Q. (2018). Potential Roles of Dental Pulp Stem Cells in Neural Regeneration and Repair. Stem Cells Int..

[172] Manni G., Buratta S., Pallotta M.T., Chiasserini D., Di Michele A., Emiliani C., Giovagnoli S., Pascucci L., Romani R., Bellezza I. (2023). Extracellular Vesicles in Aging: An Emerging Hallmark?. Cells.

[173] Park R., Spritz S., Zeng A.Y., Erukulla R., Zavala D., Merchant T., Gascon A., Jung R., Bigit B., Azar D.T. (2025). Corneal Sensory Receptors and Pharmacological Therapies to Modulate Ocular Pain. Int. J. Mol. Sci..

[174] Deeks E.D., Lamb Y.N. (2020). Cenegermin: A Review in Neurotrophic Keratitis. Drugs.

[175] Sacchetti M., Bruscolini A., Lambiase A. (2017). Cenegermin for the Treatment of Neurotrophic Keratitis. Drugs Today.

[176] Sacchetti M., Lambiase A. (2017). Neurotrophic Factors and Corneal Nerve Regeneration. Neural. Regen. Res..

[177] Tewari D., Roy A., Bavi N., Anwar K.N., Krishan A., Massoumi H., Nguyen T., Soleimani M., Xiang S., Baharnoori M. (2024). Corneal Trigeminal Nerve-Derived Extracellular Vesicles and Their Impact on Corneal Epithelial Homeostasis. Investig. Ophthalmol. Vis. Sci..

[178] Massoumi H., Amin S., Soleimani M., Momenaei B., Ashraf M.J., Guaiquil V.H., Hematti P., Rosenblatt M.I., Djalilian A.R., Jalilian E. (2023). Extracellular-Vesicle-Based Therapeutics in Neuro-Ophthalmic Disorders. Int. J. Mol. Sci..

[179] Jalilian E., Massoumi H., Bigit B., Amin S., Katz E.A., Guaiquil V.H., Anwar K.N., Hematti P., Rosenblatt M.I., Djalilian A.R. (2022). Bone Marrow Mesenchymal Stromal Cells in a 3D System Produce Higher Concentration of Extracellular Vesicles (EVs) with Increased Complexity and Enhanced Neuronal Growth Properties. Stem Cell Res. Ther..

[180] Pedersen C., Chen V.T., Herbst P., Zhang R., Elfert A., Krishan A., Azar D.T., Chang J.-H., Hu W.-Y., Kremsmayer T.P. (2024). Target Specification and Therapeutic Potential of Extracellular Vesicles for Regulating Corneal Angiogenesis, Lymphangiogenesis, and Nerve Repair. Ocul. Surf..

[181] Park M., Shin H.A., Duong V.-A., Lee H., Lew H. (2022). The Role of Extracellular Vesicles in Optic Nerve Injury: Neuroprotection and Mitochondrial Homeostasis. Cells.

[182] Massoumi H., Niktinat H., Alviar M., Guaiquil V.H., Rosenblatt M., Djalilian A.R., Jalilian E. (2025). Comparative Analysis of miRNA Profiles in Corneal and Bone Marrow Mesenchymal Stem Cells (MSC)-Derived Extracellular Vesicles (EVs): Implications for Nerve Regeneration. Investig. Ophthalmol. Vis. Sci..

[183] Arabpour Z., Niktinat H., Hatami F., Yaghmour A., Yucel Z.J., Ghalibafan S., Massoumi H., Bibak Bejandi Z., Salehi M., Jalilian E. (2025). Extracellular Vesicle (EV) Proteomics in Corneal Regenerative Medicine. Proteomes.

[184] Massoumi H., Chaudhuri M., Tewari D., Singh M., Jazayerli C., Weng S.H.S., Huff A., Soleimani M., Mohammadrashidi M., Anwar K.N. (2024). Combining Proteomics and in Vitro Approaches to Evaluate Regenerative Effect of Extracellular Vesicles from Bone Marrow and Cornea Mesenchymal Stem Cells Cultured in 2D and 3D System on Corneal Nerve Regeneration. Investig. Ophthalmol. Vis. Sci..

[185] Massoumi H., Katz E., Nguyen T.T., Zhou Q., Jazayerli C., Anwar K., Ashraf M., Soleimani M., Guaiquil V.H., Rosenblatt M. (2023). Extracellular Vesicles (EVs) from 3D Cultured Human Bone Marrow Mesenchymal Stem Cells (hBM-MSC) Demonstrated Increased Complexity and Neurite Elongation in an in-Vivo Corneal Injury Model. Investig. Ophthalmol. Vis. Sci..

[186] Samaeekia R., Rabiee B., Putra I., Shen X., Park Y.J., Hematti P., Eslani M., Djalilian A.R. (2018). Effect of Human Corneal Mesenchymal Stromal Cell-Derived Exosomes on Corneal Epithelial Wound Healing. Investig. Ophthalmol. Vis. Sci..

[187] Bazan N.G., Molina M.F., Gordon W.C. (2011). Docosahexaenoic Acid Signalolipidomics in Nutrition: Significance in Aging, Neuroinflammation, Macular Degeneration, Alzheimer’s, and Other Neurodegenerative Diseases. Annu. Rev. Nutr..

[188] Pham T.L., Bazan H.E.P. (2021). Docosanoid Signaling Modulates Corneal Nerve Regeneration: Effect on Tear Secretion, Wound Healing, and Neuropathic Pain. J. Lipid Res..

[189] Esquenazi S., Bazan H.E.P., Bui V., He J., Kim D.B., Bazan N.G. (2005). Topical Combination of NGF and DHA Increases Rabbit Corneal Nerve Regeneration after Photorefractive Keratectomy. Investig. Ophthalmol. Vis. Sci..

[190] Pham T.L., He J., Kakazu A.H., Jun B., Bazan N.G., Bazan H.E.P. (2017). Defining a Mechanistic Link between Pigment Epithelium–Derived Factor, Docosahexaenoic Acid, and Corneal Nerve Regeneration. J. Biol. Chem..

[191] Subramanian A., Krishnan U.M., Sethuraman S. (2009). Development of Biomaterial Scaffold for Nerve Tissue Engineering: Biomaterial Mediated Neural Regeneration. J. Biomed. Sci..

[192] Mastropasqua L., Nubile M., Acerra G., Detta N., Pelusi L., Lanzini M., Mattioli S., Santalucia M., Pietrangelo L., Allegretti M. (2022). Bioengineered Human Stromal Lenticule for Recombinant Human Nerve Growth Factor Release: A Potential Biocompatible Ocular Drug Delivery System. Front. Bioeng. Biotechnol..

[193] Shinta Dewi P.A., Sitompul R.S., Pawitan J.A., Naroeni A., Antarianto R.D. (2022). Improvement of Corneal Nerve Regeneration in Diabetic Rats Using Wharton’s Jelly-Derived Mesenchymal Stem Cells and Their Conditioned Medium. Int. J. Mol. Cell. Med..

[194] Wang M., Li Y., Wang H., Li M., Wang X., Liu R., Zhang D., Xu W. (2023). Corneal Regeneration Strategies: From Stem Cell Therapy to Tissue Engineered Stem Cell Scaffolds. Biomed. Pharmacother..

[195] Liu W., Deng C., McLaughlin C.R., Fagerholm P., Lagali N.S., Heyne B., Scaiano J.C., Watsky M.A., Kato Y., Munger R. (2009). Collagen–Phosphorylcholine Interpenetrating Network Hydrogels as Corneal Substitutes. Biomaterials.

[196] Fagerholm P., Lagali N.S., Merrett K., Jackson W.B., Munger R., Liu Y., Polarek J.W., Söderqvist M., Griffith M. (2010). A Biosynthetic Alternative to Human Donor Tissue for Inducing Corneal Regeneration: 24-Month Follow-Up of a Phase 1 Clinical Study. Sci. Transl. Med..

[197] Simpson F.C., McTiernan C.D., Islam M.M., Buznyk O., Lewis P.N., Meek K.M., Haagdorens M., Audiger C., Lesage S., Gueriot F.-X. (2021). Collagen Analogs with Phosphorylcholine Are Inflammation-Suppressing Scaffolds for Corneal Regeneration from Alkali Burns in Mini-Pigs. Commun. Biol..

[198] Wu M., Downie L.E., Hill L.J., Chinnery H.R. (2022). The Effect of Topical Decorin on Temporal Changes to Corneal Immune Cells after Epithelial Abrasion. J. Neuroinflamm..

[199] Kubo E., Shibata S., Shibata T., Sasaki H., Singh D.P. (2022). Role of Decorin in the Lens and Ocular Diseases. Cells.

[200] Wu M., Downie L.E., Grover L.M., Moakes R.J.A., Rauz S., Logan A., Jiao H., Hill L.J., Chinnery H.R. (2020). The Neuroregenerative Effects of Topical Decorin on the Injured Mouse Cornea. J. Neuroinflamm..

[201] Chen W., Zhang Z., Hu J., Xie H., Pan J., Dong N., Liu Z. (2013). Changes in Rabbit Corneal Innervation Induced by the Topical Application of Benzalkonium Chloride. Cornea.

[202] Vereertbrugghen A., Pizzano M., Sabbione F., Del Papa M.S., Rodríguez G., Passerini M.S., Galletti J.G. (2024). Hyaluronate Protects From Benzalkonium Chloride-Induced Ocular Surface Toxicity. Trans. Vis. Sci. Tech..

[203] Zhou T., Lee A., Lo A.C.Y., Kwok J.S.W.J. (2022). Diabetic Corneal Neuropathy: Pathogenic Mechanisms and Therapeutic Strategies. Front. Pharmacol..

[204] Lu X., Chen Z., Lu J., Watsky M.A. (2023). Effects of 1,25-Vitamin D3 and 24,25-Vitamin D3 on Corneal Nerve Regeneration in Diabetic Mice. Biomolecules.

[205] Lv W.S., Zhao W.J., Gong S.L., Fang D.D., Wang B., Fu Z.J., Yan S.L., Wang Y.G. (2015). Serum 25-Hydroxyvitamin D Levels and Peripheral Neuropathy in Patients with Type 2 Diabetes: A Systematic Review and Meta-Analysis. J. Endocrinol. Investig..

