# Supplementary material for: Aging and Corneal Nerve Health: Mechanisms of Degeneration and Emerging Therapies for the Cornea
Source: Cells. 2025 Nov 4;14(21):1730. doi: 10.3390/cells14211730 (PMC12609739; doi:10.3390/cells14211730)
Supplement: Supplementary file 1 [file cells-14-01730-s001.zip › cells-3883659-supplementary.pdf]

Aging and Corneal Nerve Health: Mechanisms of Degeneration and Emerging Therapies for Cornea

Hanieh Niktinat [1], Melinda Alviar [1], Marziyeh Kashani [1], Hamed Massoumi [1], Ali R. Djalilian [1] and Elmira Jalilian [1,2,*]

Supplementary data 1

Compound Name	Function / Mechanism	Section in Review	Figure Panel	Example Catalog Number (Vendor)
Docosahexaenoic acid (DHA)	Omega-3 PUFA; precursor to SPMs; anti-inflammatory and neuroprotective	Inflammation & Omega-3 therapies	A	D2534 (Sigma-Aldrich)
Resolvin D1 (RvD1)	Pro-resolving lipid mediator; promotes nerve repair	Inflammation & Omega-3 therapies	A	10012554 (Cayman Chemical)
Rapamycin (Sirolimus)	mTORC1 inhibitor; induces autophagy, suppresses aging pathways	Aging metabolism & mTOR signaling	B	R0395 (Sigma-Aldrich)
Metformin	AMPK activator; enhances metabolic resilience	AMPK pathway & neuroregeneration	B	D150959 (Sigma-Aldrich)
AICAR	AMPK agonist; mimics exercise-related metabolic shift	AMPK pathway & neuroregeneration	B	A9978 (Sigma-Aldrich)
Citicoline (CDP-choline)	Choline donor; supports nerve repair and function	Diabetic corneal neuropathy; topical neuroprotective therapy	C	C9756 (Sigma-Aldrich)
Vitamin B12 (Cyanocobalamin)	Neurotrophic factor; promotes epithelial and nerve recovery	Diabetic corneal neuropathy; topical neuroprotective therapy	C	V2876 (Sigma-Aldrich)
Calcitriol (1,25-dihydroxyvitamin D3)	Active Vitamin D; modulates neuroimmune and epithelial pathways	Vitamin-based neuroprotection	C	17936 (Cayman Chemical)
Retinoic Acid (All-trans)	Neural differentiation inducer in vitro	Experimental models for neural induction	D	R2625 (Sigma-Aldrich)
Forskolin	Activates cAMP pathway; promotes neurite outgrowth	Experimental models for neural induction	D	F6886 (Sigma-Aldrich)
Benzalkonium chloride (BAK)	Preservative; induces nerve and epithelial toxicity	Preservative-associated ocular neurotoxicity	E	B1383 (Sigma-Aldrich)
Timolol	Î²-blocker; potential neurotoxicity with long-term BAK exposure	Preservative-associated ocular neurotoxicity	E	T6394 (Sigma-Aldrich)
Latanoprost	Prostaglandin analog; nerve impact reported in preserved forms	Preservative-associated ocular neurotoxicity	E	L4530 (Sigma-Aldrich)
Paclitaxel	Chemotherapeutic; stabilizes microtubules; induces neurotoxicity	Systemic chemotherapy-induced nerve degeneration	F	T7402 (Sigma-Aldrich)
Oxaliplatin	Platinum agent; causes peripheral sensory nerve degeneration	Systemic chemotherapy-induced nerve degeneration	F	O9512 (Sigma-Aldrich)

Supplementary Table. Summary of Small-Molecule Compounds Discussed in This Review. This table provides an overview of the small-molecule compounds cited throughout the review, including their mechanisms of action, associated sections, and recommended figure panel groupings. Compounds are categorized as follows:

Panel A: Lipid mediators (*e.g.,* docosahexaenoic acid [DHA], resolvin D1);
Panel B: AMPK/mTOR modulators (*e.g.,* metformin, rapamycin, AICAR);
Panel C: Neurotrophic vitamins and nutrients (*e.g.,* vitamin B12, citicoline, calcitriol);
Panel D: Differentiation-inducing agents (*e.g.,* all-trans retinoic acid, forskolin);
Panel E: Ocular medications and preservatives (*e.g.,* benzalkonium chloride [BAK], timolol, latanoprost);
Panel F: Chemotherapeutic agents associated with neurotoxicity (*e.g.,* paclitaxel, oxaliplatin).
